# Mosaicism for structural non-centromeric autosomal rearrangements in disease-defined carriers: sex differences in the rearrangements profile and maternal age distributions

**DOI:** 10.1186/s13039-017-0321-9

**Published:** 2017-05-19

**Authors:** Natalia V. Kovaleva, Philip D. Cotter

**Affiliations:** 1 Academy of Molecular Medicine, Mytniskaya str. 12/44, St. Petersburg, 191144 Russian Federation; 2 Department of Pediatrics, University of California San Francisco, San Francisco, CA USA; 3 ResearchDx Inc., Irvine, CA USA

**Keywords:** Segmental somatic mosaicism, Non-centromeric autosomal rearrangement, Genomic imbalance, Sex ratio, Maternal age, Paternal age

## Abstract

**Background:**

Mosaicism for an autosomal structural rearrangement (Rea) associated with clinical manifestation of chromosomal imbalance is rare. Consequently, there is a lack of basic epidemiological characterization of this kind of mosaicism, such as population rate, cytogenetic profile of Reas involved, maternal age distribution, and sex (male to female) ratio among Rea carriers. The objectives of the present study were: (i) determination of the Rea profile in clinically affected individuals, (ii) comparative analysis of the cytogenetic profile and involvement of single chromosomes to rearrangements in affected and previously reported asymptomatic carriers, (iii) analysis of the male/female ratio in carriers of various types of Rea, and, (iv) examination of parental ages distributions according to carriers’ sex.

**Results:**

Two hundred and forty six disease-defined cases of mosaicism for autosomal non-centromeric Rea with a normal cell line of known sex were identified from the literature. There was a significant difference in single chromosome involvements compared to structural rearrangements between affected and asymptomatic carriers of unbalanced Rea, *p* =0.0030. In affected carriers, chromosome 18 was most frequently involved in structural rearrangements (12.6% of 246 instances). The least frequently rearranged were chromosomes 16 and 21 (0.8% and 1.2%, respectively). In asymptomatic carriers, the most frequently rearranged were chromosomes 5 and 21 (13% of 51 instances each). Among carriers of “loss” or “gain/loss” of genomic material, a female predominance was observed (50 M/89 F, different from population ratio of 1.06 at *p* = 0.0002). Carriers of either “gain” or balanced Rea demonstrated typical male predominance (41 M/30 F and 18 M/16 F), not different from 1.06. Maternal and paternal ages were reported in 129 and in 109 cases, respectively. There was a significant difference in maternal age distribution between male and female carriers, with mean maternal age of 25.2 years vs 28.3 years (*p* = 0.032). However, there was no difference in paternal age, with mean paternal age of 29.4 in both groups.

**Conclusion:**

The data suggested that structural rearrangements of certain chromosomes involved in mosaicism may not be tolerated by the embryo, while others have higher survival prospects. Maternal age appears to be a risk factor for somatic mosaicism of structural Rea in female offspring or might cause an adverse effect on male embryo viability.

**Electronic supplementary material:**

The online version of this article (doi:10.1186/s13039-017-0321-9) contains supplementary material, which is available to authorized users.

## Background

Somatic chromosomal mosaicism, the presence of two or more cell lines with different chromosomal constitution, is a common phenomenon in humans [1]. However, mosaicism for structural chromosomal rearrangements (N/Rea, normal line/rearrangement) is rarely reported. Consequently, there is a lack of basic epidemiological characterization of this category of mosaicism, such as population rate, cytogenetic profile of the Reas involved, maternal age distribution, and sex ratio (SR, male to female ratio) among the carriers of Reas.

Depending on factors such as the severity of genomic imbalance, the degree of mosaicism and tissue distribution, the carrier of a somatic mosaicism may be asymptomatic or may present with a variable phenotype. A recent study of patients with somatic/gonadal mosaicism described differences in cytogenetic profile among asymptomatic and affected individuals [2]. In addition, the study revealed a strong female prevalence among both affected and asymptomatic carriers of somatic/gonadal mosaicism for unbalanced Rea, unlike the typical male prevalence among carriers of balanced Rea. However, the number of affected carriers was low (2 M/10 F), not allowing for detailed evaluation of single chromosome involvement in various type of abnormalities and sex ratio among carriers of various types of Reas.

Therefore, the objectives of the present study were: (i) determination of the Rea profile in clinically affected carriers, (ii) comparative analysis of the cytogenetic profile and involvement  of single chromosomes to structural rearrangements in affected and previously reported asymptomatic carriers, (iii) analysis of the sex ratio ratio in affected carriers of various types of Rea, and (iv) examination of the effect of parental ages.

## Methods

We reviewed reports in the literature of mosaicism for N/Rea cases detected microscopically (up to 850-band level of resolution, i.e. ≥ 5 Mb), by conventional cytogenetics or by molecular cytogenetics. The cases were identified from various sources including PubMed. Reports of N/Rea affected carriers of unknown sex were excluded from the study. According to Barber [3], individuals were considered phenotypically affected when any type of phenotypic anomaly was reported, even if the etiological role of the chromosome abnormality in the same individual was questionable. From the sample collected, we further excluded cases of Rea with both breakpoints localized at pericentromeric regions, because of the strong female preponderance among carriers of such mosaicism [4, 5]. Cases of familial instability were also excluded from the study. The selection criteria was any rearrangement identified by cytogenetic or molecular cytogenetic techniques.

Two hundred and forty six cases of N/Rea, along with the data on their chromosome constitution, patient’s’s age at testing/ascertainment, parental ages at the birth of the proband, proportion of abnormal cell line(s), and the indication for testing are tabulated in Additional files 1, 2, 3, 4, 5, 6 and 7: Tables S1-S7.

Rea were classified as loss, gain, and loss/gain of genomic material. Deletions were classified as losses, duplications and additional material were classified as gains, and derivative chromosomes, isodicentrics, complex Reas, and cases with two abnormal cell lines, one of which with deletion, another one with duplication, were classified as “loss/gain”. In some instances, derivatives and other rearrangements were considered as apparent or suggestive “gain” or “loss”.

Data were analyzed using open access software listed in the Additional file 8: Table S8. References for Additional files 1, 2, 3, 4, 5, 6, 7 and 8 are listed in the Additional file 9: Supplemental References.

## Results and discussion

### N/Rea profile

A summary of the data are presented in Table 1. The prevalence of deletions over duplications in affected carriers is a well-known phenomenon. A study carried out by FISH on semen samples from control donors showed similar deletion and duplication frequencies of chromosomal regions 7q11.23, 15q11q13, and 22q11 [6] while studies on affected carriers revealed a clear excess of deletions of these regions [7, 8]. Therefore, this phenomenon may be explained by phenotypic silence of some chromosomal regions when duplicated.

**Table 1:** Cytogenetic profile of mosaic structural rearrangements

Type of rearrangement		Sex		
		Males	Females	Total
Deletions	excluding del(13) associated with retinoblastoma	20	45	65
	del(13) associated with retinoblastoma	5	8	13
Duplications		23	16	39
Rings	apparently deleted	11	15	47
	no apparent deletion	10	10	
	uncertain	1		
Unbalanced translocations	loss	1		23
	gain	6	7	
	gain/loss	3	6	
Other unbalanced rearrangements	loss		1	29
	gain	8	5	
	gain/loss	5	10	
Apparently balanced rearrangements	inversions	2	1	13
	reciprocal translocations	5	5	
Rescued rearrangements^a^	loss	1	3	17
	gain	5	2	
	gain/loss	5	1	
Total		111	136	246

^a^including 3 deletions, 2 duplications, 2 rings, 7 unbalanced translocations, and 2 other unbalanced rearrangements

Balanced rearrangement carriers among 229 affected mosaic patients (excluding 17 with rescued rearrangements) were observed at a frequency of 6%, similar to rates observed in affected non-mosaic carriers. Detailed screening of microscopically balanced *de novo* rearrangements using high-resolution genome-wide analysis detected a chromosome imbalance in 37% of patients. In 49% of these patients, the imbalances were located in one or both breakpoint regions while the others were found elsewhere in the genome [9], being therefore just coincidental or concomitant with a balanced rearrangement.

To compare the profiles in affected and asymptomatic carriers (Table 2), we excluded one abnormality with a large cohort and specific indications from the profile analysis (13 cases of interstitial del(13) associated with retinoblastoma) and sixteen rescued rearrangements because of exclusion of such cases from the previously reported group of asymptomatic carriers. Balanced Rea (reciprocal translocations and inversions) were not included in the analysis, comprising 51% of the cases in asymptomatic carriers [2]. Of the remaining 203 cases, there were 65 deletions (32%), 39 duplications (19%), 48 rings (24%), 23 unbalanced translocations (11%), and 28 other Reas (14%). There is a significant concordance of the profile of mosaic unbalanced Reas in affected carriers with the profile found in asymptomatic carriers of somatic/gonadal mosaicism, with some prevalence of deletions in the latter group. However, it should be mentioned that among affected carriers of mosaic ring chromosomes, mosaics for deleted ring chromosomes were found more frequently compared to asymptomatic carriers (55% vs 14%). Because of the small number of samples, this difference does not reach statistical significance, and additional cases are required for a conclusion.

**Table 2:** Cytogenetic profile of mosaicism for structural rearrangement in affected and asymptomatic patients

Group	No of carriers	Type of chromosome rearrangement, *n* (%)				
		Deletions^b^	Duplications	Rings	Unbalanced translocations	Other rearrangements
Affected (present study)^a^	203	65 (32%)	39 (19%)	47 (23%)^c^	23 (11%)	29 (14%)
Asymptomatic (Kovaleva, Cotter, 2016)	45	18 (40%)	9 (20%)	9 (20%)^d^	4 (9%)	5 (11%)

^a^excluding cases of apparently balanced and rescued rearrangements

^b^excluding 13 cases of del(13q) associated with retinoblastoma

^c^55% of apparently deleted rings

^d^14% of apparently deleted rings

The distribution of single chromosome across various types of rearrangements is not uniform, as summarized in Table 3. For example, chromosome 18, being the most frequent among both deleted chromosomes and rings (12 and 10 cases, respectively), is found to have no duplications. In contrast, chromosomes 1 and 12 are more frequently found to be duplicated than deleted (6 and 7 cases vs 1 and 1). Chromosomes 21 and 16 appeared to be the least subjected to rearrangements, with only 2 and 3 of 246 instances (0.8 and 1.2%, respectively).

**Table 3:** Distribution of single chromosomes according to type of rearrangements

Chromosome	Deletions^a^	Duplications	Rings	Unbalanced translocations	Other unbalanced rearrangements	Balanced rearrangements	Total
1	1	6	0	0	1	3	11
2	1	2	3	2	0	2	10
3	2	3	0	2	3	1	11
4	4	1	5	1	1	2	13
5	2	2	0	1	3	0	7
6	1	0	2	3	0	3	9
7	6	1	1	2	3	1	13
8	4	1	2	3	2	1	13
9	0	0	2	5	0	1	8
10	0	0	0	3	1	0	4
11	7	2	0	1	1	1	12
12	1	7	1	1	4	2	16
13	6	1	5	2	0	0	14
14	4	2	1	1	4	1	13
15	4	3	2	3	2	1	15
16	1	0	0	2	0	0	3
17	3	6	2	0	0	2	12
18	12	0	10	4	5	0	31
19	2	0	3	1	1	1	8
20	2	2	0	0	0	1	5
21	0	0	2	0	0	0	2
22	2	0	7	2	0	1	12
Total	65	39	48	39	31	24	246
Proportions	_0.20_0.26_0.35_	_0.11_0.16_0.23_	_0.13_0.20_0.27_	_0.11_0.16_0.23_	_0.07_0.13_0.18_	_0.06_0.10_0.16_	1,00
*P*-value	2 · 10–6						

^a^excluding 13 cases of del(13q14) associated with retinoblastoma

To compare single chromosome involvement to structural rearrangements between affected and asymptomatic carriers, we have removed balanced rearrangements (Table 4). There is a statistically significant difference between the groups at *p* =0.0030. Such analysis is of potential meaning for evaluation of fitness of mosaic preimplantation embryos. It might be possible that rearrangements of certain chromosomes (for example, deletion of chromosome 18) are not tolerated by the embryo while others, being involved in segmental mosaicism (for example, chromosomes 5 and 21), might have good prospects. However, again, more cases should be collected for such study. Ultimately, lethality would be a function of critical genetic content. Genotype-phenotype comparisons are more complicated in mosaic cases, compounded by the level of mosaicism and the tissue distribution.

**Table 4:** Distribution of single chromosomes in affected and asymptomatic patients

Affected^a^	Asymptomatic^b^
8	
8	2
10	3
12	3
8	7 (13%)
6	1
13	
12	3
7	
4	1
11	1
14	1
14	3
12	3
14	3
3	1
11	3
31 (14%)	2 (4%)
7	1
4	3
2	7 (13%)
11	3
222	51
Difference between groups is statistically significant, *p* = 0.0030	

^a^excluding balanced rearrangements (translocations and inversions)

^b^excluding 13 cases of del(13q14) associated with retinoblastoma

### Frequency of detection of somatic N/Rea mosaicism

The data suggests that somatic mosaicism may be more frequent than expected: 3 mosaics were detected among 32 carriers of del(5) (q14) when examining at least 125 metaphases in each individual [10]. 2 of 16 cytogenetically visible 11p13 deletions and 3 cryptic 11p13 deletions were mosaic [11]. Of 27 patients with del(16) (p11.2), two were mosaics [12], and 25 mosaics were detected among 126 del(13) (q14) reports [13]. Cytogenetic analysis showed a del(15) (q11-13) in 12 patients in whom the clinical diagnosis was certain; in two there was mosaicism, and one patient also had a t(7;15) translocation [14]. In 17 cases of monosomy of 18q12.3 one was a mosaic with a normal cell line [15]. Among 29 carriers of ring chromosomes, three had a normal cell line [16], and among six patients with a r(22), one was mosaic for a normal cell line [17]. In 1966–1991, 10 patients with r(18) were diagnosed among 82,000 patients karyotyped for constitutional reasons; three of these 10 presented with mosaicism for normal line [18].

A recent study on the frequency of mosaicism for balanced Rea, showed that mosaicism for inversions was the most common (3/103 = 2.9%) followed by mosaicism for reciprocal translocations (7/453 = 1.5%), while mosaicism for Robertsonian translocations was the least common (2/265 = 0.8%) [2]. These data obtained from the analysis of studies on a total of 56,760 patients with reproductive failures were consistent with corresponding data from a report on a constitutional chromosome analysis in 74,306 consecutive patients [19].

Diagnosis of whole chromosome or structural Rea mosaicism is likely under-reported due to low level mosaicism. Sciorra et al. [20] reported that it is the policy of most clinical genetic laboratories to count only 15 or 20 cells and to analyze 2 or 3 metaphases for work up of patients. This laboratory approach is due to various time and financial constraints, as well as *the assumption that mosaicism for a structural rearrangement, while theoretically possible, is an unlikely event*”. However, the data accumulated in the literature indicated that mosaicism might be more frequent than recognized currently.

### Parental and cell origin of N/Rea mosaicism

The parental origin of chromosomes involved in mosaic rearrangements was reported in few cases, being paternal in six instances [21–25] and maternal in three instances [25–27]. Additionally, the paternal origin of the abnormal chromosome was reported in two carriers of gonadal mosaicism [28, 29], yielding to cumulative figures of eight paternally derived rearrangements vs three maternally derived rearrangements. Theoretically, if mosaicism arises mostly post-zygotically, then paternally and maternally derived rearrangements are expected to have equal frequency. However, at present, there is still insufficient data to allow any certain conclusions on whether there is a bias in parental origin of postzygotic rearrangements.

The preferential paternal origin was reported for various types of *de novo* non-mosaic unbalanced structural rearrangements: Wolf-Hirshchorn syndrome-associated rearrangements [30], del 18p- [31], del 22q13.3 [32], and a preponderance of paternally derived deletions 5p14 were reported in two studies [33, 34]. In addition, the majority of *de novo* cytogenetically balanced reciprocal translocations are of paternal origin [35]. Preferential formation in the paternal germline were detected for *de novo* balanced complex chromosome Reas [36]. High rates of *de novo* 15q11q13 inversions was found in human spermatozoa [37]. A recent study using array comparative genome hybridization confirmed a significant paternal bias for *de novo* structural variations by any mechanism in 118 individuals with intellectual disability [38].

Mosacism with the presence of a normal cell line is commonly assumed to result from postzygotic errors. However, there is evidence that such rearrangements may originate during meiosis or may be inherited. In such cases, there should be postzygotic rescue events leading to formation of normal cell lines. Recently, Robberecht et al. [25] demonstrated that two of nine cases with mosaic segmental structural imbalances (>15%) resulted from meiotic errors, followed by multiple parallel trisomy rescue events.

Cases of proved or presumptive rescued rearrangements together with a formation of normal cell line are summarized in Additional file 7: Table S7. The majority of the mosaics for inherited Rea were not evaluated for chimerism. However, as there was sex concordance between normal and abnormal cell lines the presence of chimerism is unlikely; in chimerism some cases would be expected to be sex discordant. In addition, chimerism is an extremely rare event.

An unusual finding was the comparatively high rate of involvement of chromosome 11 in rescue events, with four cases of ten. Moreover, in one of them, the rescue appeared to be familial since the mother was a mosaic for the same abnormality with UPD for a deleted region [39]. Apparent familial tendency to rescue was reported by Juberg et al. [40] who described two sibs with mosaicism for a paternally transmitted abnormality.

All cases of mosaicism for *de novo* rearrangements had been evaluated to investigate the mechanisms of the rearrangement formation (Additional file 7: Table S7). The parental origin of the rearranged chromosome resulted from a paternal meiotic error in four cases [25, 41–43] and maternal in three cases [24, 44, 45].

### Male to female ratio

The male to female ratio was analyzed across various types of rearrangements presented in Table 1 and showed significant variation depending on the type of Rea. Female predominance was observed in carriers of either “loss” or “gain/loss” Rea (50 M/89 F, different from population ratio of 1.06 at *p* = 0.0002. Carriers of either apparent “gain” or apparent balanced Rea (including rings without apparent deletion) demonstrated absence of female predominance (41 M/30 F and 18 M/16 F, respectively) not statistically different from population ratio of 1.06.

A recent study reported a strong female predominance among asymptomatic carriers of somatic/gonadal mosaicism for unbalanced Rea. Since no distortion in sex ratio was found among carriers of mosaicism for balanced Rea, a male-specific selection against abnormal cells in early embryo development was proposed [2]. However, results from the present study might indicate that selection against abnormal cells, if it exists, depends on the type of the Rea and the size of genomic imbalance. Apparently, duplications resulting in gains of chromosomal material, if not occurring more frequently in males, are not selected against in males. Similarly, both loss and gain/loss, if not occurring more frequently in females, are more tolerated in females than in males. Again, such unexpected and intriguing findings require further study.

### Parental ages

**Fig. 1:**
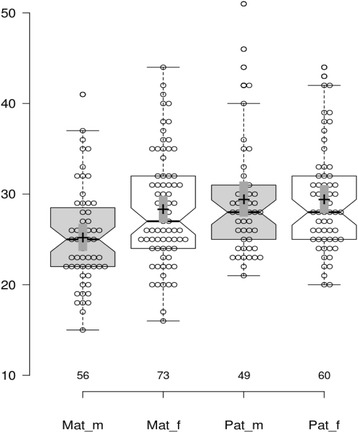
Notched boxplots for the ages of mothers (*Mat*) and fathers (*Pat*) of males (*m*) and females (*f*). Numbers at the x-axis are sample sizes

**Fig. 2:**
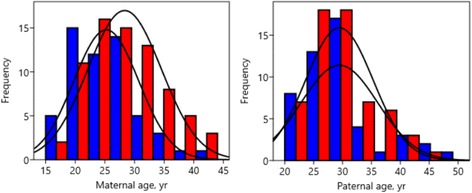
Collation of histograms for the age of mothers of males (*blue*) and females (*red*)

Maternal and paternal ages were reported in 129 and 109 cases, respectively. Surprisingly we have identified a difference in maternal age distributions between male and female carriers, with mean maternal age of 25.2 years (95% CL 23.8–26.6) vs 28.3 years (95% CL 26.9–29.7), respectively, the difference is significant at *p* = 0.032. However, there is no difference in paternal age, with mean age of 29.4 years in both male and female carriers (see Figs. 1 and 2). Sex ratio displays an apparent tendency to decrease with increase of maternal age, from 4.7 in the group of <20 year to 0.3 in the group of aged 40 year and older (Table 5). No such trend was found when analyzing sex ratio according to paternal ages.

**Table 5:** Sex ratio in patients with segmental mosaicism according to maternal age

Age groups, years	Males		Females		
	Number	Proportion (P1)	Number	Proportion (P2)	Sex ratio (P1/P2)
<20	8	0.14	2	0.03	4.7
20–24	19	0.34	18	0.25	1.4
25–29	19	0.34	24	0.33	1.0
30–34	5	0.09	14	0.19	0.5
35–39	4	0.07	10	0.14	0.5
≥40	1	0.02	5	0.07	0.3
Total	56	1.0	73	1.0	

We are not aware of any previous publications reporting maternal age distribution differences between carriers of segmental mosaicism in relation to carriers’ sex. This unexpected finding suggests an effect of age-related factors either on the postzygotic stability of female genomes or on intrauterine selection against affected male embryos, or on the male-specific selection against abnormal cells in early embryo development. A high intrauterine lethality of male carriers is less likely because of a lack of male predominance among abortuses with segmental mosaicism (Kovaleva, unpublished). In addition, in the group of apparently balanced carriers with no sex ratio distortion,, there is a difference between males’ and females’ maternal ages (23.3 years vs 30.8 years), similar to that in groups with predominance of female carriers.

Additional studies on male to female ratios in prenatally diagnosed individuals and in preimplantation embryos would clarify this issue. Further studies on the origin and mechanisms of formation of mosaicism for structural chromosomal abnormalities are indicated.

## Conclusions

The cytogenetic profile of phenotype-associated Reas (responsible for abnormal clinical features) shows a predominance for deletions of genetic material. Rearrangements of certain chromosomes may not be tolerated by the embryo while others, being involved in segmental mosaicism, might have a more favorable prospects. A significant female prevalence among carriers of mosaicism for loss of genomic material, as well as among carriers of mosaicism for both loss and gain of genomic material, suggests either a male-specific selection against abnormal cell line(s) or reduced viability of male fetuses. The absence of a skewed sex ratio in carriers of mosaicism for gain of genomic material may indicate that gains, despite being disease-causing, are tolerated in proliferating cells of male embryos unlike losses of genomic material. Maternal age might be a risk factor of occurrence of somatic mosaicism for structural Rea in female offspring or might cause an adverse effect on male embryo viability. Further evaluation of parental and cell origin of mosaic Rea would be advisable for elucidation of these intriguing subjects.

## Additional files

Additional file 1:Table S1. Mosaicism for deletions. Tabular data presenting details of affected carriers of mosaicism for deletion: karyotype, parental ages at patient's birth, patient’s age at ascertainment/testing, proportion of abnormal cell line(s), and indications for cytogenetic testing. (XLSX 12 kb)

Additional file 2: Table S2. Mosaicism for duplications. Tabular data presenting details of affected carriers of mosaicism for duplication: karyotype, parental ages at patient's birth, patient’s age at ascertainment/testing, proportion of abnormal cell line(s), and indications for cytogenetic testing. (XLSX 8 kb)

Additional file 3: Table S3. Mosaicism for ring chromosomes. Tabular data presenting details of affected carriers of mosaicism for ring chromosome: karyotype, parental ages at patient's birth, patient’s age at ascertainment/testing, proportion of abnormal cell line(s), and indications for cytogenetic testing. (XLSX 10 kb)

Additional file 4: Table S4. Mosaicism for unbalanced translocations. Tabular data presenting details of affected carriers of mosaicism for unbalanced translocation: karyotype, parental ages at patient's birth, patient’s age at ascertainment/testing, proportion of abnormal cell line(s), and indications for cytogenetic testing. (XLSX 9 kb)

Additional file 5: Table S5. Mosaicism for other unbalanced rearrangements. Tabular data presenting details of affected carriers of mosaicism for other unbalanced rearrangement: karyotype, parental ages at patient's birth, patient’s age at ascertainment/testing, proportion of abnormal cell line(s), and indications for cytogenetic testing. (XLSX 7 kb)

Additional file 6: Table S6. Mosaicism for apparently balanced rearrangements. Tabular data presenting details of affected carriers of mosaicism for apparently balance translocation or inversion: karyotype, parental ages at patient's birth, patient’s age at ascertainment/testing, proportion of abnormal cell line(s), and indications for cytogenetic testing. (XLSX 6 kb)

Additional file 7: Table S7. Mosaicism due to rescued rearrangement. Tabular data presenting details of affected carriers of mosaicism for rescued rearrangement: karyotype, parental ages at patient's birth, patient’s age at ascertainment/testing, proportion of abnormal cell line(s), indications for cytogenetic testing, and description of method(s) of confirmatory study. (XLSX 7 kb)

Additional file 8: Table S8. Software used for the statistical data analysis. List of the programmes used for the data analysis, programme titles, version and/or date of release, URL, and references. (DOCX 17 kb)

Additional file 9: Reference list for Tables S1-S8. (DOCX 76 kb)
