# Supplementary material for: Mosaicism for structural non-centromeric autosomal rearrangements in disease-defined carriers: sex differences in the rearrangements profile and maternal age distributions
Source: Mol Cytogenet. 2017 May 19;10:18. doi: 10.1186/s13039-017-0321-9 (PMC5438540; doi:10.1186/s13039-017-0321-9)
Supplement: Supplementary file 8 — Software used for the statistical data analysis. List of the programmes used for the data analysis, programme titles, version and/or date of release, URL, and references. (DOCX 17 kb) [file 13039_2017_321_MOESM8_ESM.docx]

TABLE SVIII. Software used for the statistical data analysis

| Program | Version and/or date | Intention | URL | Reference |
| --- | --- | --- | --- | --- |
| BoxPlotR | 23.07.2014 | Notched boxplot generation | http://boxplot.tyerslab.com/ |  |
| PAST | 3.1  02.2016 | Point and interval estimations, normality tests, significance testing using bootstrap and Monte Carlo and cluster analysis | http://folk.uio.no/  ohammer/past/ | Hammer et al., 2001 |
| JASP | 0.7.5.5 | Bayesian *t*-test and Cohen’s *d*_C_ estimation | https://jasp-stats.org/ | Lowe et al., 2015 |
| BF Calculators |  | Bayes factor for grouped or two-sample *t*-tests | http://pcl.missouri.edu/  bayesfactor | Rouder et al., 2009 |
| G*Power | 3.1.9.2 28.03.2014 | Power analysis | http://www.gpower.hhu.de/ | Faul et al., 2007 |
| LePrep | 2.1.0  01.04.2011 | Probabilities of replication and prediction intervals | http://lmrs.univ-rouen.fr/  Persopage/Lecoutre/PAC.htm |  |
| Reference Value Advisor | 2.1  09.12.2015 | Computing reference intervals using the standard and robust methods | http://www.biostat.envt.fr/  spip/spip.php?article63 | Geffré et al., 2011 |
| StatXact | 8 | 150 Tests and Procedures for Exact Inference | http://www.cytel.com/software/statxact |  |
