# Supplementary material for: Mosaicism for structural non-centromeric autosomal rearrangements in disease-defined carriers: sex differences in the rearrangements profile and maternal age distributions
Source: Mol Cytogenet. 2017 May 19;10:18. doi: 10.1186/s13039-017-0321-9 (PMC5438540; doi:10.1186/s13039-017-0321-9)
Supplement: Supplementary file 9 — Reference list for Tables S1-S8. (DOCX 76 kb) [file 13039_2017_321_MOESM9_ESM.docx]

**SUPPLEMENTAL REFERENCES**

Al-Hassnan ZN, Faden M, Al-Owain M, Iqbat M. De novo mosaic 46,XX,dup(11)(q13q25)/46,XX in a patient with trigonocephaly. Eur J Hum Genet. 2005;13 Suppl 1:164.

Alp MY, Cebi AH, Seyhan S, Cansu A, Ikbal M. A case of de novo mosaic 18q21.3 deletion with a mild phenotype. Genet Couns. 2014;25:71-3.

Altunoğlu U, Karaman B, Basaran S, Kayserili H. Girl with left hemiatrophy reveals confined mosaicisms for r(13) in fibroblasts. Eur J Hum Genet. 2009;17 Suppl 2:125.

Al-Zahrani J, Al-Dosari N, AbuDheim N, Alshidi TA, Colak D, Al-Habit O, Al-Odaib A, Sakati N, Meyer B, Ozand PT, Kaya N. Chromosome 12q24.31-q24.33 deletion causes multiple dysmorphic features and developmental delay: First mosaic patient and overview of the phenotype related to 12q24qter defects. Mol Cytogenet. 2011;4:9.

Amouri A, Ayed W, Bhouri R, El Kamel-Lebbi I, Kilani O, Guermani H, Abidli N, Talmoudi F, Abdelhak S, Bouayed-Abdelmoula N. Clinical findings and cytogenetic analysis of a ring chromosome 7 in a girl referred for suspicion of Fanconi anaemia. Eur J Hum Genet. 2009;17 Suppl 2:130.

Anderson CE, Wallerstein R, Zamerowski ST, Witzleben C, Hoyer JR, Gibas L, Jackson LG. Ring chromosome 4 mosaicism coincidence of oligomeganephronia and signs of Seckel syndrome. Am J Med Genet. 1997;72:281-5.

Andria G, Melis D, Perone L, Sperandeo MP, Passariello A, Sibilio M, Sebastio G. Is mosaicism at the origin of the extreme variability of 13q deletion phenotype? Amer J Hum Genet. 2001;61 Suppl 4:705.

Antonenko VG, Levina LYa, Solonichenko VG. A case of mosaicism for partial trisomy of chromosome 8 long arm resulted from t(7;8)(p22;q11). In: Kuleshov NP, Lourie IW, editors. Current problems in clinical cytogenetics, Moskow: 1991. p. 29-30.

[Antonini S](http://www.ncbi.nlm.nih.gov/pubmed/?term=AntoniniS%5BAuthor%5D&cauthor=true&cauthor_uid=12407704), [Kim CA](http://www.ncbi.nlm.nih.gov/pubmed/?term=KimCA%5BAuthor%5D&cauthor=true&cauthor_uid=12407704), [Sugayama SM](http://www.ncbi.nlm.nih.gov/pubmed/?term=SugayamaSM%5BAuthor%5D&cauthor=true&cauthor_uid=12407704), [Vianna-Morgante AM](http://www.ncbi.nlm.nih.gov/pubmed/?term=Vianna-MorganteAM%5BAuthor%5D&cauthor=true&cauthor_uid=12407704). Delimitation of duplicated segments and identification of their parental origin in two partial chromosome 3p duplications. [Am J Med Genet](http://www.ncbi.nlm.nih.gov/pubmed/12407704). 2002;113:144-50.

Aughton DJ, Al Saadi AA, Canady AI, Lucas BM. Balanced reciprocal translocation mosaicism associated with an abnormal phenotype. Am J Med Genet. 1993. 45:721-4.

Babaran S, Kayserili H, Keser Y, Lüleci G, Jüksel M. Three further cases of duplication 3q. Eur J Hum Genet. 1966;4 Suppl 1:31.

Back E, Voiculescu I, Brunger M, Wolff G. Familial ring (20) chromosomal mosaicism. Hum Genet. 1989;83:148-54.

Badalian LO, Dement'eva GM, Malygina NA, Mutovin GR, Petrukhin AS. Mosaic variant of the translocation form of syndrome 18q-. Genetika. 1981;17:1867-71.

[Bagherizadeh](http://www.ncbi.nlm.nih.gov/pubmed/?term=BagherizadehE%5Bauth%5D) E, [Behjati](http://www.ncbi.nlm.nih.gov/pubmed/?term=BehjatiF%5Bauth%5D) F, [Saberi](http://www.ncbi.nlm.nih.gov/pubmed/?term=SaberiSH%5Bauth%5D) SH, [Shafeghati](http://www.ncbi.nlm.nih.gov/pubmed/?term=ShafeghatiY%5Bauth%5D) Y. Prenatal diagnosis in a mentally retarded woman with mosaic ring chromosome 18. Indian J Hum Genet. 2011;17:111–3.

Bamfort JS, Lin CC. DK phocomelia phenotype (von Voss-Cherstvoy syndrome) caused by somatic mosaicism for del(13q). Am J Med Genet. 1997;73:408-11.

Berkheim LK, Lewin SA, Issa B, Drozd-Borydiuk A, Meloni-Ehrig A, Mao R,Brothman AR, Carey JC. Mosaicism for deletion of 15q11.2q13 in a patient with atypical Angelman syndrome phenotype: a second case. Am J Hum Genet. 2002;71 Suppl 4:622.

Bint SM, Davies AF, Ogilvie CM. Multicolor banding remains an important adjunct to array CGH and conventional karyotyping. Mol Cytogenet. 2013;6:55.

Black S, Smith J. Structural chromosome mosaicism associated with craniosynostosis, good growth and development. Am J Hum Genet. 1985;37 Suppl 4:46A.

Blouin JL, Aurias A, Creau-Goldberg N, Apiou F, Alcaide-Loridan C, Bruel A, Prieur M, Kraus J, Delabar JM, Sinet PM. Cytogenetic and molecular analysis of a de novo tandem duplication of chromosome 21. Hum Genet. 1991;88:167-74.

Bonaglia MC, Giorda R, Beri S, Bigoni S, Sense A, Baroncini A, Capucci A, De Agostini C, Gwilliam R, Deloukas P, Dunham I, Zuffardi O. Mosaic 22q13 deletions: evidence for concurrent mosaic segmental isodisomy and gene conversion. Eur J Hum Genet. 2009;17:426-33.

Borgaonkar DS. 1990. Repository of human chromosomal variants and anomalies. 13^th^ Listing. Med Center of Delaware and Univ of Delaware, Newark, Delaware, pp.874.

Bryke CR, Lindgren V, Fryburg JS, Yang-Feng TL. Novel isodicentric chromosome 18 in an abnormal infant with a mosaic karyotype [46,XY/46,XY,-18,+dic(18)(q12.2]). Am J Med Genet. 1990;36:247-50.

[Bueno I](http://www.ncbi.nlm.nih.gov/pubmed/?term=Bueno%20I%5BAuthor%5D&cauthor=true&cauthor_uid=3579058), [Olivares JL](http://www.ncbi.nlm.nih.gov/pubmed/?term=Olivares%20JL%5BAuthor%5D&cauthor=true&cauthor_uid=3579058), [Olmedillas MJ](http://www.ncbi.nlm.nih.gov/pubmed/?term=Olmedillas%20MJ%5BAuthor%5D&cauthor=true&cauthor_uid=3579058), [Abad M](http://www.ncbi.nlm.nih.gov/pubmed/?term=Abad%20M%5BAuthor%5D&cauthor=true&cauthor_uid=3579058), [Bueno M](http://www.ncbi.nlm.nih.gov/pubmed/?term=Bueno%20M%5BAuthor%5D&cauthor=true&cauthor_uid=3579058). Terminal partial mosaic monosomy of the short arm of chromosome 3, in discordant monozygotic twins, 46,XY/46,XY, del (3) (p25)]. [An Esp Pediatr](http://www.ncbi.nlm.nih.gov/pubmed/3579058). 198;26:187-90.

[Bühler EM](http://www.ncbi.nlm.nih.gov/pubmed/?term=BühlerEM%5BAuthor%5D&cauthor=true&cauthor_uid=3594935), [Bühler UK](http://www.ncbi.nlm.nih.gov/pubmed/?term=BühlerUK%5BAuthor%5D&cauthor=true&cauthor_uid=3594935), [Beutler C](http://www.ncbi.nlm.nih.gov/pubmed/?term=BeutlerC%5BAuthor%5D&cauthor=true&cauthor_uid=3594935), [Fessler R](http://www.ncbi.nlm.nih.gov/pubmed/?term=FesslerR%5BAuthor%5D&cauthor=true&cauthor_uid=3594935). A final word on the tricho-rhino-phalangeal syndromes. [Clin Genet](http://www.ncbi.nlm.nih.gov/pubmed/?term=clin+genet+1987+31+273). 1987;31:273-5.

[Bunin GR](http://www.ncbi.nlm.nih.gov/pubmed/?term=BuninGR%5BAuthor%5D&cauthor=true&cauthor_uid=2915374), [Meadows AT](http://www.ncbi.nlm.nih.gov/pubmed/?term=MeadowsAT%5BAuthor%5D&cauthor=true&cauthor_uid=2915374), [Buckley JD](http://www.ncbi.nlm.nih.gov/pubmed/?term=BuckleyJD%5BAuthor%5D&cauthor=true&cauthor_uid=2915374), [Woods WG](http://www.ncbi.nlm.nih.gov/pubmed/?term=WoodsWG%5BAuthor%5D&cauthor=true&cauthor_uid=2915374), [Hammond GD](http://www.ncbi.nlm.nih.gov/pubmed/?term=HammondGD%5BAuthor%5D&cauthor=true&cauthor_uid=2915374). Frequency of 13q abnormalities among 203 patients with retinoblastoma. [J Natl Cancer Inst](http://www.ncbi.nlm.nih.gov/pubmed/?term=J+natl+cancer+inst+1989+81+370). 1989;81:370-4.

[Canonero I](http://www.ncbi.nlm.nih.gov/pubmed/?term=CanoneroI%5BAuthor%5D&cauthor=true&cauthor_uid=22760760), [Montes C](http://www.ncbi.nlm.nih.gov/pubmed/?term=MontesC%5BAuthor%5D&cauthor=true&cauthor_uid=22760760), [Sturich A](http://www.ncbi.nlm.nih.gov/pubmed/?term=SturichA%5BAuthor%5D&cauthor=true&cauthor_uid=22760760), [Boterón M](http://www.ncbi.nlm.nih.gov/pubmed/?term=BoterónM%5BAuthor%5D&cauthor=true&cauthor_uid=22760760), [Asinari M](http://www.ncbi.nlm.nih.gov/pubmed/?term=AsinariM%5BAuthor%5D&cauthor=true&cauthor_uid=22760760), [Cuestas E](http://www.ncbi.nlm.nih.gov/pubmed/?term=CuestasE%5BAuthor%5D&cauthor=true&cauthor_uid=22760760), [Rossi N](http://www.ncbi.nlm.nih.gov/pubmed/?term=RossiN%5BAuthor%5D&cauthor=true&cauthor_uid=22760760). [Phelan McDermid syndrome: five patients description and report on the first case described in conjoined twins]. [Arch Argent Pediatr](http://www.ncbi.nlm.nih.gov/pubmed/22760760). 2012;110:e50-54.

[Cantu JM, Rivas F, Ruiz C, Barajas LO, Moller M, Rivera H.](http://www.ncbi.nlm.nih.gov/entrez/query.fcgi?cmd=Retrieve&db=pubmed&dopt=Abstract&list_uids=3879442&query_hl=1) Trisomy 7p due to a mosaic normal/dir dup(7)(p13----p22). Syndrome delineation, critical segment assignment, and a comment on duplications. Ann Genet. 1985;28:254-7.

Cantu ES, Thomas IT, Frias JL. Unusual cytogenetic mosaicism involving chromosome 14 abnormalities in a child with an MR/MCA syndrome and abnormal pigmentation. Clin Genet. 1989;36:189-95.

Carreira IM, Melo JB, Rodrigues C, Back L, Vermeesch J, Weise A, Kosyakova N, Oliveira G, Matoso E. Molecular cytogenetic characterisation of a mosaic add(12)(p13.3) with an inv dup(3)(q26.31 → qter) detected in an autistic boy. Mol Cytogenet. 2009;2:16.

Caspersson T, Hulten M, Lindsten J, Zech L. 1971. Chromatid interchange resulting in duplication of the short arm of chromosome number 7 in man. Ann Genet. 14:143-144.

Clark RD, Fenner-Gonzales M. Apparent Fryns syndrome in a boy with a tandem duplication. Am J Med Genet. 1989;34:422-6.

Collins FA, Maclean K, Wu ZH, Malafiej P, Darmanian A, Daniel A, Peters G. “Phylloid” pattern skin mosaicism in a 15 month old girl with multiple congenital malformations and mosaicism for partial monosomy 13 [46,XX/46,XX,-13,+r(13)]. Am J Hum Genet. 2002;71 Suppl 4:592.

Concolino D, Moricca MT, Jembo MA, Apa R, Caloiero D, Saullo E, Strisciuglio P. Ring chromosome 9: clinical and cytogenetics characterization. Am J Hum Genet. 2002;71 Suppl 4:593.

Corona-Rivera A, Bobadilla-Morales L, Mendoza-Becerra S, Carbajal-Lopez F, Barros-Ninez P, Corona-Rivera JR. Ring chromosome case report and cell cycle studies. Am J Hum Genet. 2003;73 Suppl 5:302.

Cotter PD, Hirshhorn K. Chimerism detected by an unbalanced chromosome translocation: an alternative hypothesis. Clin Genet. 1998;53:230.

Crosley CJ, Mitter NS, Gardner LI. Mosaic deletion 17p: a mild expression of Miller-Dieker syndrome. Am J Hum Genet. 1986;39 Suppl 3:A58.

Cummins JH, Surti U, Mowery-Rushton P, Bay CA. Mosaic partial trisomy 2p: completion of chromosomal characterization 17 years after initial visit. Am J Hum Genet. 1997;61Suppl 4:A372.

Daniel A, Malafiej P, Preece K, Nelson J, Smith M. Identification of marker chromosomes in thirteen patients using FISH probing. Am J Med Genet. 1994;53:8-18.

Daniel A, St Heaps L, Sylvester D, Diaz S, Peters G. Two mosaic terminal inverted duplications arising post-zygotically: evidence for possible formation of neo-telomeres. Cell & Chromosome. 2008;7:1.

Davies AF, Kirby TL, Docherty Z, Mackie Ogilvie C. 2003. Characterization of terminal chromosome anomalies using multisubtelomere FISH. Am J Med Genet 120A:483-489.

Degtyareva ZM, Vorsanova SG. 1991. Case of mosaic syndrome 13q-. In: Kuleshov NP, Lourie IW, editors. Current problems in clinical cytogenetics, Moskow: 1991. p. 47.

de Silva D, Massie D, Drummond J, Couzin D, Dean JC. Mosaicism for a tandem duplication dup(1)(q12q22) in an 18 year old female. J Med Genet. 1998;35:600-3.

[Digilio MC](http://www.ncbi.nlm.nih.gov/pubmed/?term=DigilioMC%5BAuthor%5D&cauthor=true&cauthor_uid=8014974), [Giannotti A](http://www.ncbi.nlm.nih.gov/pubmed/?term=GiannottiA%5BAuthor%5D&cauthor=true&cauthor_uid=8014974), [Floridia G](http://www.ncbi.nlm.nih.gov/pubmed/?term=FloridiaG%5BAuthor%5D&cauthor=true&cauthor_uid=8014974), [Uccellatore F](http://www.ncbi.nlm.nih.gov/pubmed/?term=UccellatoreF%5BAuthor%5D&cauthor=true&cauthor_uid=8014974), [Mingarelli R](http://www.ncbi.nlm.nih.gov/pubmed/?term=MingarelliR%5BAuthor%5D&cauthor=true&cauthor_uid=8014974), [Danesino C](http://www.ncbi.nlm.nih.gov/pubmed/?term=DanesinoC%5BAuthor%5D&cauthor=true&cauthor_uid=8014974), [Dallapiccola B](http://www.ncbi.nlm.nih.gov/pubmed/?term=DallapiccolaB%5BAuthor%5D&cauthor=true&cauthor_uid=8014974), [Zuffardi O](http://www.ncbi.nlm.nih.gov/pubmed/?term=ZuffardiO%5BAuthor%5D&cauthor=true&cauthor_uid=8014974). Trisomy 8 syndrome owing to isodicentric 8p chromosomes: regional assignment of a presumptive gene involved in corpus callosum development. [J Med Genet](http://www.ncbi.nlm.nih.gov/pubmed/8014974). 1994;31:238-41.

Dixon JW, Costa T, Teshima IE. Mosaicism for duplication 12q (12q13-->q24.2) in a dysmorphic male infant. J Med Genet. 1993;30:70-2.

Dudin G, Nasr A, Traboulsi E, Khouri F, Der Kaloustian VM. 1984. Hereditary retinoblastoma and 13q--mosaicism. Cytogenet Cell Genet 38:235-237.

Dvornichenko NS, Samovarov VV, Tkacheva TM. Mosaic deletion and inversion of chromosome 11. Ultrasound Perinat Diagn (Ukraine). 2007;NN23-24:84.

Engelen JJ, De Die-Smulders CE, Back E. De novo mosaic 46,XX,del(3)(q21q25)/46,XX karyotype in a patient with BPES. Genet Couns. 2002;13:359-61.

Erez A, Li J, Geraghty MT, Ben-Shachar S, Cooper ML, Mensing DE, Vonalt KD, Ou Z, Pursley AN, Chinault AC, Patel A, Cheung SW, Sahoo T. Mosaic deltion 11p13 in a child with dopamine beta-hydroxylase deficiency – case report and review of the literature. Am J Med Genet. 2010;152A:732-6.

Eugster EA, Berry SA, Hirsch B. Mosaicism for deletion 1p36.33 in a patient with obesity and hyperphagia. Am J Hum Genet 1997;70:409-12.

Faivre L, Viot G, Prieur M, Turleau C, Gosset P, Romana S, Munnich A, Vekemans M, Cormier-Daire V. Apparent Sotos syndrome (cerebral gigantism) in a child with trisomy 20p11.2-p12.1 mosaicism. Am J Med Genet. 2000 ;91:273-6.

Faul F, Erdfelder E, Lang A-G, Buchner AG. Power 3: A flexible statistical power analysis program for the social, behavioral, and biomedical sciences. Behav Res Methods. 2007;39:175-91.

Finucane BM, Kurtz MB, Babu VR, Scott CI, Jr. Mosaicism for deletion 17p11.2in a boy with the Smith-Magenis syndrome. Am J Med Genet. 1993;45:447-9.

Floore C, Robertson A, Samuel I, Williamson N, McLeod DR, Hoganson G, Hoo JJ. A pseudoisochromosome 18q and an isodicentric chromosome 18. Clin Genet. 1989;35:450-4.

Fryns JP, Petit P, Heffnick R, van den Berghe H. Mosaic pericentric inversion of chromosome 2. J Genet Hum. 1983;31:157-61.

Fryns JP, Lambrechts A, Jansseune H, Van den Berghe H. Moderate mental retardation and nonspecific dysmorphic syndrome associated with ring chromosome 9. Hum Genet. 1979;50:29-32.

Fryns JP, Kleczkowska A, Jaeken J, Van den Berghe H. Ring chromosome 4 mosaicism and Potter sequence. Ann Genet. 1988;31:120-2.

Fryns JP, Kleczkowska A, van den Berghe H. Ring chromosome 6: twenty years follow-up. Ann Genet. 1990;33:179.

Fryns JP, Kleczkowska A. Autism and ring chromosome 18 mosaicism. Clin Genet. 1992;42:55.

Fryns JP, Kleczkowska A, Decock P, Massa G, van den Berghe H. 46,XX/46,XX,del(20)(pter-->p12.2) mosaicism limited to fibroblasts associated with MCA/MR and severe growth deficit. Ann Genet. 1992;35:234-6.

Fujiwara M, Kamasaki H, Morita Y, Kamada M. A mosaic case of isodicentric chromosome 18. Acta Paediatr Jpn. 1992;34:534-8.

Galvin JA, LeBoyer RM, Michelotti M, Del Monte MA, Elner VM, Mian SI. Mosaic chromosome 18q deletion syndrome with bilateral full-thickness corneal disease: surgical intervention and histopathology. Ophtalmic Genet. 2015;36:75-8.

[Gamage TH](http://www.ncbi.nlm.nih.gov/pubmed/?term=GamageTH%5BAuthor%5D&cauthor=true&cauthor_uid=22754240), [Godapitiya IU](http://www.ncbi.nlm.nih.gov/pubmed/?term=GodapitiyaIU%5BAuthor%5D&cauthor=true&cauthor_uid=22754240), [Nanayakkara S](http://www.ncbi.nlm.nih.gov/pubmed/?term=NanayakkaraS%5BAuthor%5D&cauthor=true&cauthor_uid=22754240), [Jayasekara RW](http://www.ncbi.nlm.nih.gov/pubmed/?term=JayasekaraRW%5BAuthor%5D&cauthor=true&cauthor_uid=22754240), [Dissanayake VH](http://www.ncbi.nlm.nih.gov/pubmed/?term=DissanayakeVH%5BAuthor%5D&cauthor=true&cauthor_uid=22754240). A child with mosaicism for deletion (14)(q11.2q13). [Indian J Hum Genet](http://www.ncbi.nlm.nih.gov/pubmed/?term=Indian+j+hum+genet+2012+18+130). 2012;18:130-3.

Garcia-Rodriguez E, Garcia-Garcia E, Perez-Sanchez A, Pavon-Delgado A. A new observation of 13q deletion syndrome: severe undescribed features. Genet Couns. 2015;26:213-7.

Gardner LI, Mitter NS, Welsh-Sloan J, Miller R. Mosaic partial trisomy 9q13->qter in a boy [46,XY/46,XY,-8,+der(8)t(8;9)(q11;q13). Am J Hum Genet. 1985;37 Suppl 4:A53.

Gardner RJ, Dockery HE, Fitzgerald PH, Parfitt RG, Romain DR, Scobie N, Shaw RL, Tumewu P, Watt AJ. Mosaicism with a normal cell line and an autosomal structural rearrangement. J Med Genet. 1994;31:108-14.

Garnica A, Muneer RS, Hopcus D, Ruth H, McCampbell K, Lynch J. An unusual tissue specific normal/recombinant 11 chromosomal mosaicism in a male infant. Am J Hum Genet. 1989;45 Suppl 4:A76.

Geffré A, Concordet D, Braun JP, Trumel C. Reference Value Advisor: a new freeware set of macroinstructions to calculate reference intervals with Microsoft Excel. Veterinary Clinical Pathology. 2011;40:107-12.

Genovese MJ, Krawczun MS, Jenkins EC, Stark-Houck SL, Sklower-Brooks SL, Raguthu S, Wisniewski K. Blood/skin mosaicism for chromosomal aberration. Am J Hum Genet. 1991;49 Suppl:283A.

Germain-Lee EL, Schiffman G, Mules EH, Lederman HM. Selective deficiency of antibody responses to polysaccharide antigens in a child mosaic for partial trisomy 1 (46,XX,dir dup (1) (q12----q23)/46,XX). J Pediatr. 1990;117 Pt 1:96-9.

Gijsbers ACJ, Dauwerse JG, Bosch CAJ, Boon EMJ, van den Ende W, Kant SG, Hansson KMB, Breuning MH, Bakker E, Ruivenkamp CAL. Three new cases with mosaicism involving a normal cell line and a cryptic unbalanced autosomal reciprocal translocation. Eur J Med Genet. 2011;54:e409-12.

Goossens E, Decock P, Potgieters S, Fryns JP. Mosaic/normal15q11-q13 duplication associated with developmental delay but normal phenotype. Genet Couns. 1999;10:133-6.

Gordon K, Siu VM, Sergovich F, Jung J. 18q- mosaicism associated with Rett syndrome. Am J Med Genet. 1993;46:142-4.

Gradek GA, Kvistad PH, Houge G. Monosomy rescue gave cells with normal karyotype in a mildly affected man with 46,XY,r(8) mosaicism. Eur J Med Genet. 2006;49:292-7.

Grass F., Brown C, Layton A, Ostrowski R, Spence J. Ring chromosome 4: cryptic mosaicism, karyotypic evolution and phenotypic effect. Am J Hum Genet. 1998;63 Suppl 4:A136.

Guilherme R, Klein E, Hamid A, Bhatt S, Volleth M, Polityko A, Kulpanovich A, Dufke A, Albrecht B, Morlot S, Brecevic L, Petersen M, Manolakos E, Kosyakova N, Liehr T. Human ring chromosomes – new insights for their clinical significance. Balkan J Med Genet. 2013;16:13-20.

[Guilherme RS](http://www.ncbi.nlm.nih.gov/pubmed/?term=GuilhermeRS%5BAuthor%5D&cauthor=true&cauthor_uid=24700634), [Soares KC](http://www.ncbi.nlm.nih.gov/pubmed/?term=SoaresKC%5BAuthor%5D&cauthor=true&cauthor_uid=24700634), [Simioni M](http://www.ncbi.nlm.nih.gov/pubmed/?term=SimioniM%5BAuthor%5D&cauthor=true&cauthor_uid=24700634), [Vieira TP](http://www.ncbi.nlm.nih.gov/pubmed/?term=VieiraTP%5BAuthor%5D&cauthor=true&cauthor_uid=24700634), [Gil-da-Silva-Lopes VL](http://www.ncbi.nlm.nih.gov/pubmed/?term=Gil-da-Silva-LopesVL%5BAuthor%5D&cauthor=true&cauthor_uid=24700634), [Kim CA](http://www.ncbi.nlm.nih.gov/pubmed/?term=KimCA%5BAuthor%5D&cauthor=true&cauthor_uid=24700634), [Brunoni D](http://www.ncbi.nlm.nih.gov/pubmed/?term=BrunoniD%5BAuthor%5D&cauthor=true&cauthor_uid=24700634), [Spinner NB](http://www.ncbi.nlm.nih.gov/pubmed/?term=SpinnerNB%5BAuthor%5D&cauthor=true&cauthor_uid=24700634), [Conlin LK](http://www.ncbi.nlm.nih.gov/pubmed/?term=ConlinLK%5BAuthor%5D&cauthor=true&cauthor_uid=24700634), [Christofolini DM](http://www.ncbi.nlm.nih.gov/pubmed/?term=ChristofoliniDM%5BAuthor%5D&cauthor=true&cauthor_uid=24700634), [Kulikowski LD](http://www.ncbi.nlm.nih.gov/pubmed/?term=KulikowskiLD%5BAuthor%5D&cauthor=true&cauthor_uid=24700634), [Steiner CE](http://www.ncbi.nlm.nih.gov/pubmed/?term=SteinerCE%5BAuthor%5D&cauthor=true&cauthor_uid=24700634), [Melaragno MI](http://www.ncbi.nlm.nih.gov/pubmed/?term=MelaragnoMI%5BAuthor%5D&cauthor=true&cauthor_uid=24700634). Clinical, cytogenetic, and molecular characterization of six patients with ring chromosomes 22, including one with concomitant 22q11.2 deletion. [Am J Med Genet](http://www.ncbi.nlm.nih.gov/pubmed/24700634). 2014;164A:1659-65.

Hajanpor AK, Habibian R, Wohlmuth C. Leiomyoma of uterus in a patient with ring chromosome 12: case presentation and literature review. Am J Med Genet. 1996;63:335-9.

Hamers AJH, van Kempen C. Ring chromosome 8 in a boy with multiple congenital abnormalities and mental retardation. J Med Genet. 1977;14:451-5.

Hammer Ø, Harper DAT, Ryan PD. PAST: Paleontological statistics software package for education and data analysis. Palaeontol Electron. 2001;4:9pp.

Hansson K, Ruivenkamp C, Gijsbers A, Rövenkamp-Abels L, Smit L, Laurense-Bik M, Heijboer K, Kriek M, Koopmans M, Smit LME. Mosaic interstitial duplication 14q. Eur J Hum Genet. 2012;20 Suppl 1:115.

Hansson KBM, Ruivenkamp CAL, Szuhai K, Hoogenboom ME, Knepfle C, Segboer T, Kant S, den Hollander NS. Mosaic interstitial duplication 17q. Eur J Hum Genet. 2011;19 Suppl 2:146.

Harrod MJ, Byrne JB, Dev VG, Francke U. Duplication 12q mosaicism in two unrelated patients with a similar syndrome. Am J Med Genet. 1980;7:123-9.

Hata A, Suzuki Y, Matsui I, Kuroki Y. Ring 18 mosaicism in identical twins. Hum Genet. 1982;62:364-7.

Helmuth RA, Weaver DD, Wills ER. Holoprosencephaly, ear abnormalities, congenital heart defect, and microphallus in a patient with 11q- mosaicism. Am J Med Genet. 1989;32:178-81.

[Herva R](http://www.ncbi.nlm.nih.gov/pubmed/?term=HervaR%5BAuthor%5D&cauthor=true&cauthor_uid=7389182), [Vuorinen O](http://www.ncbi.nlm.nih.gov/pubmed/?term=VuorinenO%5BAuthor%5D&cauthor=true&cauthor_uid=7389182). Congenital heart disease with del(15q) mosaicism. Clin Genet. 1980;17:26-8.

[Hervé B](http://www.ncbi.nlm.nih.gov/pubmed/?term=HervéB%5BAuthor%5D&cauthor=true&cauthor_uid=25963108), [Roume J](http://www.ncbi.nlm.nih.gov/pubmed/?term=RoumeJ%5BAuthor%5D&cauthor=true&cauthor_uid=25963108), [Cognard S](http://www.ncbi.nlm.nih.gov/pubmed/?term=CognardS%5BAuthor%5D&cauthor=true&cauthor_uid=25963108), [Fauvert D](http://www.ncbi.nlm.nih.gov/pubmed/?term=FauvertD%5BAuthor%5D&cauthor=true&cauthor_uid=25963108), [Molina-Gomes D](http://www.ncbi.nlm.nih.gov/pubmed/?term=Molina-GomesD%5BAuthor%5D&cauthor=true&cauthor_uid=25963108), [Vialard F](http://www.ncbi.nlm.nih.gov/pubmed/?term=VialardF%5BAuthor%5D&cauthor=true&cauthor_uid=25963108). Low-level mosaicism of a de novo derivative chromosome 9 from a t(5;9)(q35.1;q34.3) has a major phenotypic impact. [Eur J Med Genet](http://www.ncbi.nlm.nih.gov/pubmed/25963108). 2015;58:346-50.

Hickman G, Küpferling P. Dysmorphia syndrome with 13q- mosaic. Clin Genet. 1985;28:436.

[Hirshfeld AB](http://www.ncbi.nlm.nih.gov/pubmed/?term=HirshfeldAB%5BAuthor%5D&cauthor=true&cauthor_uid=11343316), [Thompson WR](http://www.ncbi.nlm.nih.gov/pubmed/?term=ThompsonWR%5BAuthor%5D&cauthor=true&cauthor_uid=11343316), [Patel A](http://www.ncbi.nlm.nih.gov/pubmed/?term=PatelA%5BAuthor%5D&cauthor=true&cauthor_uid=11343316), [Boone LB](http://www.ncbi.nlm.nih.gov/pubmed/?term=BooneLB%5BAuthor%5D&cauthor=true&cauthor_uid=11343316), [Murphy AM](http://www.ncbi.nlm.nih.gov/pubmed/?term=MurphyAM%5BAuthor%5D&cauthor=true&cauthor_uid=11343316). Proximal trisomy of 1q mosaicism in a girl with hypertrophic cardiomyopathy associated with Wolff-Parkinson-White syndrome and multiple congenital anomalies. [Am J Med Genet](http://www.ncbi.nlm.nih.gov/pubmed/11343316). 2001;100:264-8.

Hoang S, Ahn J, Manna R, Binta S, Mansourc S, Homfrayc T, Mohammedd S, Ogilvie CM. Detection of mosaicism for genome imbalance in a cohort of 3,042 clinical cases using an oligonucleotide array CGH platform. Eur J Med Genet. 2010;54:121-9.

Horn D, Happle R, Neitzel H, Kunze J. Pigmentary mosaicism of the hyperpigmented type in two half-brothers. Am J Med Genet. 2002;112:65-9.

Hou JW, Liu CH, Wang TR, Zhu HM, Jiang S, Sciorra LJ, Lee ML. Mosaic ring chromosome 13 analyzed by fluorescence in situ hybridization: report of a case. J Formos Med Assoc. 1992;91:1108-11.

Il’ina EG, Gurevich DB. A case of mosaicism for del(14)(q13->qter). The Register of chromosomal anomalies. Moskow: Institute of Medical Genetics; 1984. p. 148.

Jacobs PA, Melville M, Ratcliffe S, Keay AJ, Syme J. A cytogenetic survey of 11860 newborn infants. Ann Hum Genet. 1974;37:359-76.

Jacobs PA, Matsuura JS, Mayer M, Newlands IM. A cytogenetic survey of an institution for the mentally retarded: I. Chromosome abnormalities. Clin Genet. 1978;13:37-60.

Jobanputra V, Wilson A, Shirazi M, Feenstra H, Levy B, Anyane-Yeboa K, Warburton D. Partial uniparental disomy with mosaic deletion 13q in an infant with multiple congenital anomalies. Am J Med Genet. 2013;161A:2393-5.

Johnson JP, Haag M, Beischel L, McCann C, Phillips S, Tunby M, Hansen J, Schwanke C, Reynolds JF. ‘Deletion rescue’ by mitotic 11q uniparental disomy in a family with recurrence of 11q deletion Jacobsen syndrome. Clin Genet. 2013;85:376-80.

Jotterand V, Boisjoly HM, Harnois C, Bigonesse P, Laframboise R, Gagne R, St-Pierre A. 11p13 Deletion, Wilms' tumour, and aniridia: unusual genetic, non-ocular and ocular features of three cases. Br J Ophthalmol. 1990;74:568-70.

Juberg RC, Stallard R, Mowrey P, Valido C L. Dissociation of a t(12;21) resulting in a normal cell line in two trisomic 21 sons of a nonmosaic t(12;21) father? Hum Genet. 1983;64:216-21.

Judge CG, Garson OM, Pitt DB, Sutherland GR. A girl with Wolf-Hirschhorn syndrome and mosaicism 46,XX/46,XX,4p-. J Ment Defic Res. 1974;18:79-85.

Kapitanova N, Ciuladaite Z, Burnyte B, Utkus A. Clinical characterization of a patient with mosaic microdeletion 7q36.1-qter. Eur J Hum Genet. 2014;22 Suppl 1:450.

Karamysheva TV, Matveeva VG, Shorina AP, Rubtsov NB. Clinical and molecular cytogenetic analysis of a rare case of mosaicism for partial monosomy 3p and partial trisomy 10q in human. Genetika. 2001;37:811-6.

Keng WT, Harewood L, Grace E, Paxton C, Lam WW, Fitzpatrick DR. A balanced translocation in a case of hypomelanosis of Ito with confirmation of mosaicism using buccal cell interphase FISH. Am J Med Genet. 2006;140:1111-3.

Khlybova GP, Tsybel VI, Kuleshov NP, Zaletayev DV. 1991. A case of ring chromosome 22. In: Kuleshov NP, Lourie IW, editors. Current problems in clinical cytogenetics, Moskow: 1991. p. 68.

Kitsiou S, Kolialexi A, Mavrou A. Mosaic Cri du Chat syndrome in a patient exhibiting three 5p cell lines. Prenat Diagn. 2004;24:578-9.

[Kivelä T](http://www.ncbi.nlm.nih.gov/pubmed/?term=KiveläT%5BAuthor%5D&cauthor=true&cauthor_uid=14522775)1, [Tuppurainen K](http://www.ncbi.nlm.nih.gov/pubmed/?term=TuppurainenK%5BAuthor%5D&cauthor=true&cauthor_uid=14522775), [Riikonen P](http://www.ncbi.nlm.nih.gov/pubmed/?term=RiikonenP%5BAuthor%5D&cauthor=true&cauthor_uid=14522775), [Vapalahti M](http://www.ncbi.nlm.nih.gov/pubmed/?term=VapalahtiM%5BAuthor%5D&cauthor=true&cauthor_uid=14522775). Retinoblastoma associated with chromosomal 13q14 deletion mosaicism. [Ophthalmology](http://www.ncbi.nlm.nih.gov/pubmed/?term=kivela+and+riikonen). 2003;110:1983-8.

Kleczkowska A, Fryns JP, Van den Berghe H. On the variable effect of mosaic normal/balanced chromosomal rearrangements in man. J Med Genet. 1990;27:505-7.

[Kotzot D](http://www.ncbi.nlm.nih.gov/entrez/query.fcgi?db=pubmed&cmd=Search&term=), [Martinez MJ](http://www.ncbi.nlm.nih.gov/entrez/query.fcgi?db=pubmed&cmd=Search&term=), [Bagci G](http://www.ncbi.nlm.nih.gov/entrez/query.fcgi?db=pubmed&cmd=Search&term=), [Basaran S](http://www.ncbi.nlm.nih.gov/entrez/query.fcgi?db=pubmed&cmd=Search&term=), [Baumer A](http://www.ncbi.nlm.nih.gov/entrez/query.fcgi?db=pubmed&cmd=Search&term=), [Binkert F](http://www.ncbi.nlm.nih.gov/entrez/query.fcgi?db=pubmed&cmd=Search&term=), [Brecevic L](http://www.ncbi.nlm.nih.gov/entrez/query.fcgi?db=pubmed&cmd=Search&term=), [Castellan C](http://www.ncbi.nlm.nih.gov/entrez/query.fcgi?db=pubmed&cmd=Search&term=), [Chrzanowska K](http://www.ncbi.nlm.nih.gov/entrez/query.fcgi?db=pubmed&cmd=Search&term=), [Dutly F](http://www.ncbi.nlm.nih.gov/entrez/query.fcgi?db=pubmed&cmd=Search&term=), [Gutkowska A](http://www.ncbi.nlm.nih.gov/entrez/query.fcgi?db=pubmed&cmd=Search&term=), [Karauzum SB](http://www.ncbi.nlm.nih.gov/entrez/query.fcgi?db=pubmed&cmd=Search&term=), [Krajewska-Walasek M](http://www.ncbi.nlm.nih.gov/entrez/query.fcgi?db=pubmed&cmd=Search&term=), [Luleci G](http://www.ncbi.nlm.nih.gov/entrez/query.fcgi?db=pubmed&cmd=Search&term=), [Miny P](http://www.ncbi.nlm.nih.gov/entrez/query.fcgi?db=pubmed&cmd=Search&term=), [Riegel M](http://www.ncbi.nlm.nih.gov/entrez/query.fcgi?db=pubmed&cmd=Search&term=), [Schuffenhauer S](http://www.ncbi.nlm.nih.gov/entrez/query.fcgi?db=pubmed&cmd=Search&term=), [Seidel H](http://www.ncbi.nlm.nih.gov/entrez/query.fcgi?db=pubmed&cmd=Search&term=), [Schinzel A](http://www.ncbi.nlm.nih.gov/entrez/query.fcgi?db=pubmed&cmd=Search&term=). Parental origin and mechanisms of formation of cytogenetically recognisable de novo direct and inverted duplications. J Med Genet. 2000;37:281-6.

Kotzot D, Rothlisberger B, Riegel M, Schinzel A. Maternal uniparental isodisomy 11q13->qter in a dysmorphic and mentally retarded female with partial trisomy mosaicism 11q13->qter. J Med Genet. 2001;38:876-81.

Krabchi K, Lavoie J, Laframboise R, Bronsard M, Drouin R. Cytogenetic and molecular studies of a de novo mosaic trisomy 12p. Am J Hum Genet. 2000;67 Suppl 4:781.

Kroisel PM, Petek E, Wagner K. Skin pigmentary anomalies in a mosaic form of partial tetrasomy 3q. J Med Genet. 2000;37:723-5.

Kucinskas V, Sliuzac V, Utkus A, Cimbalistiene L. Mosaic chromosome 21 abnormality in the patients with syndromic lip and cleft palate. Chrom Res. 2005;13 Suppl 1:41.

Kulharya AS, Lovell CM, Flannery DB. Unusual mosaic karyotype resulting from adjacent 1 segregation of t(11;22): importance of performing skin fibroblast karyotype in patients with unexplained multiple congenital anomalies. Am J Med Genet. 2002;113:367-70.

Lacassie Y, Cortés F, Molina V, Montgomery AH. Discordant phenotype due to ring 13 chromosomal mosaic syndrome in one identical twin. Am J Hum Genet. 1993 ;53 Suppl 3:571.

Larkins SA, Davison EV, Raafat F, Buick RG, Goodman A, Mann JR. A case of Wilms’tumour associated with mosaicism for an interstitial deletion of 11p with no other features of WAGR complex. Eur J Hum Genet. 1996;4 Suppl 1:150.

[Lazjuk GI](http://www.ncbi.nlm.nih.gov/entrez/query.fcgi?db=pubmed&cmd=Search&term=), [Lurie IW](http://www.ncbi.nlm.nih.gov/entrez/query.fcgi?db=pubmed&cmd=Search&term=), [Kravtzova GI](http://www.ncbi.nlm.nih.gov/entrez/query.fcgi?db=pubmed&cmd=Search&term=), [Usoev SS](http://www.ncbi.nlm.nih.gov/entrez/query.fcgi?db=pubmed&cmd=Search&term=). New cytogenetic variant of Orbeli's syndrome (46,XY-45,XY,-D-46,XY,Dq+). Humangenetik. 1973;20:219-21.

Levy J, Receveur A, Jedraszak G, Chantot-Bastaraud S, Renaldo F, Gondry J, Copin H, Siffroi J-P, Portnoï MF. Involvement of interstitial telomeric sequences in two new cases of mosaicism for autosomal structural rearrangements. Am J Med Genet. 2015;167A:428-33.

Lejeune J, Berger R, Réthoré MO, Lafour-Cade J, Dutrillaux B, Canlorbe P, Labrune B. Deux cas de syndrome 18q- en mosaique (46,XX/46,XX,18q-). Ann Genet. 1967;10:18-24.

L[iberfarb RM](http://www.ncbi.nlm.nih.gov/pubmed/?term=LiberfarbRM%5BAuthor%5D&cauthor=true&cauthor_uid=6521998), [Bustos T](http://www.ncbi.nlm.nih.gov/pubmed/?term=BustosT%5BAuthor%5D&cauthor=true&cauthor_uid=6521998), [Miller WA](http://www.ncbi.nlm.nih.gov/pubmed/?term=MillerWA%5BAuthor%5D&cauthor=true&cauthor_uid=6521998), [Sang D](http://www.ncbi.nlm.nih.gov/pubmed/?term=SangD%5BAuthor%5D&cauthor=true&cauthor_uid=6521998). Incidence and significance of a deletion of chromosome band 13q14 in patients with retinoblastoma and in their families. [Ophthalmology](http://www.ncbi.nlm.nih.gov/pubmed/?term=liberfarb+rm+and+bustos).  1984;91:1695-9.

Lo-Castro A, El-Malhany, Galasso C, Verrotti A, Nardone AM, Postorivo D, Palmieri C, Curatolo P. De novo mosaic ring chromosome 18 in a child with mental retardation, epilepsy and immunological problems. Eur J Med Genet. 2011;54: e329-32.

Lorentz CP, Jalal SM, Thompson DM, Babovic-Vuksanovic D. Mosaic r(13) resulting in large deletion of chromosome 13q in a newborn female with multiple congenital anomalies. Am J Med Genet. 2002;111:61-7.

Lowe J, Selker R, Verhagen J, Marsman M, Gronau QF, Jamil T, Smira M, Epskamp S, Wild A, Ly A, Matzke D, Wagenmakers E-J, Morey RD, Rouder JN. [Software to Sharpen Your Stats](http://www.psychologicalscience.org/index.php/publications/observer/2015/march-15/bayes-or-bust-with-new-softwares.html). Observer. 2015;28(3).

Mack J, Nimmakayalu M, Pober B, Qumsiyeh MB. Mapping of a mosaic deletion of the short arm of chromosome 7 (7p15.3 to 7p21.1). Amer J Hum Genet. 2001;69 Suppl 4:811.

[Manolakos E](http://www.ncbi.nlm.nih.gov/pubmed/?term=ManolakosE%5BAuthor%5D&cauthor=true&cauthor_uid=19014423), [Kosyakova N](http://www.ncbi.nlm.nih.gov/pubmed/?term=KosyakovaN%5BAuthor%5D&cauthor=true&cauthor_uid=19014423), [Thomaidis L](http://www.ncbi.nlm.nih.gov/pubmed/?term=ThomaidisL%5BAuthor%5D&cauthor=true&cauthor_uid=19014423), [Neroutsou R](http://www.ncbi.nlm.nih.gov/pubmed/?term=NeroutsouR%5BAuthor%5D&cauthor=true&cauthor_uid=19014423), [Weise A](http://www.ncbi.nlm.nih.gov/pubmed/?term=WeiseA%5BAuthor%5D&cauthor=true&cauthor_uid=19014423), [Mihalatos M](http://www.ncbi.nlm.nih.gov/pubmed/?term=MihalatosM%5BAuthor%5D&cauthor=true&cauthor_uid=19014423), [Orru S](http://www.ncbi.nlm.nih.gov/pubmed/?term=OrruS%5BAuthor%5D&cauthor=true&cauthor_uid=19014423), [Kokotas H](http://www.ncbi.nlm.nih.gov/pubmed/?term=KokotasH%5BAuthor%5D&cauthor=true&cauthor_uid=19014423), [Kitsos G](http://www.ncbi.nlm.nih.gov/pubmed/?term=KitsosG%5BAuthor%5D&cauthor=true&cauthor_uid=19014423), [Liehr T](http://www.ncbi.nlm.nih.gov/pubmed/?term=LiehrT%5BAuthor%5D&cauthor=true&cauthor_uid=19014423), [Petersen MB](http://www.ncbi.nlm.nih.gov/pubmed/?term=PetersenMB%5BAuthor%5D&cauthor=true&cauthor_uid=19014423). Complex chromosome rearrangement in a child with microcephaly, dysmorphic facial features and mosaicismfor a terminal deletion del(18)(q21.32-qter) investigated by FISH and array-CGH: Case report. [Mol Cytogenet](http://www.ncbi.nlm.nih.gov/pubmed/19014423). 2008;1:24.

Marcus-Soekarman D, Hamers G, Velzeboer S, Nijhuis J, Loneus WH, Herbergs J, de Die-Smulders C, Schrander-Stumpel C, Engelen J. Mosaic trisomy 11p in monozygotic twins with discordant clinical phenotypes. Am J Med Genet. 2004;124A:288-91.

[Martínez A](http://www.ncbi.nlm.nih.gov/pubmed/?term=MartínezA%5BAuthor%5D&cauthor=true&cauthor_uid=18402336), [Ramos S](http://www.ncbi.nlm.nih.gov/pubmed/?term=RamosS%5BAuthor%5D&cauthor=true&cauthor_uid=18402336), [González-del Angel A](http://www.ncbi.nlm.nih.gov/pubmed/?term=González-delAngelA%5BAuthor%5D&cauthor=true&cauthor_uid=18402336), [Alcántara MA](http://www.ncbi.nlm.nih.gov/pubmed/?term=AlcántaraMA%5BAuthor%5D&cauthor=true&cauthor_uid=18402336), [Molina B](http://www.ncbi.nlm.nih.gov/pubmed/?term=MolinaB%5BAuthor%5D&cauthor=true&cauthor_uid=18402336), [Carnevale A](http://www.ncbi.nlm.nih.gov/pubmed/?term=CarnevaleA%5BAuthor%5D&cauthor=true&cauthor_uid=18402336).   Duplication 2p and monosomy 8p in mosaicism: clinical, molecular cytogenetic and molecular markers of a unique case. [Rev Invest Clin](http://www.ncbi.nlm.nih.gov/pubmed/18402336). 2007;59:444-8.

[Melis D](http://www.ncbi.nlm.nih.gov/pubmed/?term=MelisD%5BAuthor%5D&cauthor=true&cauthor_uid=16317301), [Pia Sperandeo M](http://www.ncbi.nlm.nih.gov/pubmed/?term=PiaSperandeoM%5BAuthor%5D&cauthor=true&cauthor_uid=16317301), [Perone L](http://www.ncbi.nlm.nih.gov/pubmed/?term=PeroneL%5BAuthor%5D&cauthor=true&cauthor_uid=16317301), [Staiano A](http://www.ncbi.nlm.nih.gov/pubmed/?term=StaianoA%5BAuthor%5D&cauthor=true&cauthor_uid=16317301), [Andria G](http://www.ncbi.nlm.nih.gov/pubmed/?term=AndriaG%5BAuthor%5D&cauthor=true&cauthor_uid=16317301), [Sebastio G](http://www.ncbi.nlm.nih.gov/pubmed/?term=SebastioG%5BAuthor%5D&cauthor=true&cauthor_uid=16317301). Mosaic 13q13.2-ter deletion restricted to tissues of ectodermal and mesodermal origins. [Clin Dysmorphol](http://www.ncbi.nlm.nih.gov/pubmed/16317301). 2006;15:13-8.

Mikelsaar R, Klassman A. Mosaic “pure” interstitial deletion del(7)(q32q34). Eur J Hum Genet. 1996;4 Suppl 1:34.

Mikelsaar R, Klassman A. Mosaic interstitial deletion del(15)(q22q24). Medizinishe Genetik. 1997;9:299.

Mikelsaar RV, Varb K, Suvari A, Schinzel A. Mosaic terminal del(19)(q13.33:) in a girl with seizures and mental retardation. J Med Genet. 2001;38:e2.

Mikelsaar R, Roomets E, Pihlau E. New syndrome – chromosome 19q distal deletion syndrome. Eur J Hum Genet. 2004;12 Suppl 1:138.

Miller BA, Jayakar P, Capo H. Child with multiple congenital anomalies and mosaicism 46, XX/46,XX, del (14)(q32.3). Am J Med Genet. 1992 ;44:635-7.

Motegi T, Ichikawa A, Noda M, Hashimoto G, Kaga M. 18p-Mosaicism: case report and review. Hum Genet. 1978;44:213-7.

Motegi T. High rate of detection of 13q14 deletion mosaicism among retinoblastoma patients (using more extensive methods). Hum Genet. 1982;61:95-7.

Motegi T, Minoda K. A decreasing tendency for cytogenetic abnormality in peripheral lymphocytes of retinoblastoma patients with 13q14 deletion mosaicism. Hum Genet. 1984;66:186-9.

[Mounoud RL](http://www.ncbi.nlm.nih.gov/entrez/query.fcgi?db=pubmed&cmd=Search&term=), [Klein D](http://www.ncbi.nlm.nih.gov/entrez/query.fcgi?db=pubmed&cmd=Search&term=), [Bettschart W](http://www.ncbi.nlm.nih.gov/entrez/query.fcgi?db=pubmed&cmd=Search&term=), [Cabrol C](http://www.ncbi.nlm.nih.gov/entrez/query.fcgi?db=pubmed&cmd=Search&term=). A clinical and cytogenetic investigation carried out in a special institution for mentally retarded patients: preliminary results concerning 82 cases of oligophrenia. J Genet Hum. 1976;24:297-335.

[Mul D](http://www.ncbi.nlm.nih.gov/pubmed/?term=MulD%5BAuthor%5D&cauthor=true&cauthor_uid=22214923), [Wu S](http://www.ncbi.nlm.nih.gov/pubmed/?term=WuS%5BAuthor%5D&cauthor=true&cauthor_uid=22214923), [de Paus RA](http://www.ncbi.nlm.nih.gov/pubmed/?term=dePausRA%5BAuthor%5D&cauthor=true&cauthor_uid=22214923), [Oostdijk W](http://www.ncbi.nlm.nih.gov/pubmed/?term=OostdijkW%5BAuthor%5D&cauthor=true&cauthor_uid=22214923), [Lankester AC](http://www.ncbi.nlm.nih.gov/pubmed/?term=LankesterAC%5BAuthor%5D&cauthor=true&cauthor_uid=22214923), [Duyvenvoorde HA](http://www.ncbi.nlm.nih.gov/pubmed/?term=DuyvenvoordeHA%5BAuthor%5D&cauthor=true&cauthor_uid=22214923), [Ruivenkamp CA](http://www.ncbi.nlm.nih.gov/pubmed/?term=RuivenkampCA%5BAuthor%5D&cauthor=true&cauthor_uid=22214923), [Losekoot M](http://www.ncbi.nlm.nih.gov/pubmed/?term=LosekootM%5BAuthor%5D&cauthor=true&cauthor_uid=22214923), [Tol MJ](http://www.ncbi.nlm.nih.gov/pubmed/?term=TolMJ%5BAuthor%5D&cauthor=true&cauthor_uid=22214923), [De Luca F](http://www.ncbi.nlm.nih.gov/pubmed/?term=DeLucaF%5BAuthor%5D&cauthor=true&cauthor_uid=22214923), [van de Vosse E](http://www.ncbi.nlm.nih.gov/pubmed/?term=vandeVosseE%5BAuthor%5D&cauthor=true&cauthor_uid=22214923), [Wit JM](http://www.ncbi.nlm.nih.gov/pubmed/?term=WitJM%5BAuthor%5D&cauthor=true&cauthor_uid=22214923). A mosaic de novo duplication of 17q21-25 is associated with GH insensitivity, disturbed in vitro CD28-mediated signaling, and decreased STAT5B, PI3K, and NF-κB activation. [Eur J Endocrinol](http://www.ncbi.nlm.nih.gov/pubmed/22214923). 2012;166:743-52.

Munier F, Pescia G, Jotterand-Bellomo M, Balmer A, Gailloud C, Thonney F. Constitutional karyotype in retinoblastoma. Case report and review of literature. Ophthalmic Paediatr Genet. 1986;10:129-50.

Nasiri F, Mahjoubi F, Soleimani S, Rahnama M, Mortezapour F, Manouchehn F, Razazian F, Zamanian M. Chromosomal findings in 8727 Iranian patients with mental retardation. Eur J Hum Genet. 2007;15 Suppl 1:115.

Naylor EW, Murphey WH, Domoszlai EI, Guthrie R. Erythropoietic protoporphyria, heterozygous cystinuria, and reduced peptidase A activity in a patient with 46,XX/46,XX,18q--mosaicism. J Med Genet. 1978;15:157-60.

Ness GO, Lybaek H, Houge G. Usefulness of high-resolution comparative genomic hybridization (CGH) for detecting and characterizing constitutional chromosome abnormalities. Am J Med Genet. 2002;113:125-36.

Nielsen J, Homma A, Rasmussen K, Ried E, Sorensen K, Saldana-Garcia P. Deletion 14q and pericentric inversion 14. J Med Genet. 1978;15:236-8.

Nyberg RH, Haapala AK, Simola KOJ. A case of human chimerism detected by unbalanced chromosomal translocation. Clin Genet. 1992;42: 257-9.

[Oliveira G](http://www.ncbi.nlm.nih.gov/pubmed/?term=OliveiraG%5BAuthor%5D&cauthor=true&cauthor_uid=12757357), [Matoso E](http://www.ncbi.nlm.nih.gov/pubmed/?term=MatosoE%5BAuthor%5D&cauthor=true&cauthor_uid=12757357), [Vicente A](http://www.ncbi.nlm.nih.gov/pubmed/?term=VicenteA%5BAuthor%5D&cauthor=true&cauthor_uid=12757357), [Ribeiro P](http://www.ncbi.nlm.nih.gov/pubmed/?term=RibeiroP%5BAuthor%5D&cauthor=true&cauthor_uid=12757357), [Marques C](http://www.ncbi.nlm.nih.gov/pubmed/?term=MarquesC%5BAuthor%5D&cauthor=true&cauthor_uid=12757357), [Ataíde A](http://www.ncbi.nlm.nih.gov/pubmed/?term=AtaídeA%5BAuthor%5D&cauthor=true&cauthor_uid=12757357), [Miguel T](http://www.ncbi.nlm.nih.gov/pubmed/?term=MiguelT%5BAuthor%5D&cauthor=true&cauthor_uid=12757357), [Saraiva J](http://www.ncbi.nlm.nih.gov/pubmed/?term=SaraivaJ%5BAuthor%5D&cauthor=true&cauthor_uid=12757357), [Carreira I](http://www.ncbi.nlm.nih.gov/pubmed/?term=CarreiraI%5BAuthor%5D&cauthor=true&cauthor_uid=12757357). Partial tetrasomy of chromosome 3q and mosaicism in a child with autism. [J Autism Dev Disord](http://www.ncbi.nlm.nih.gov/pubmed/12757357).  2003;33:177-85.

Orye E, Benoit Y, Coppieters R, Jeannin P, Vercruysse C, Delaey J, Delbeke MJ. A case of retinoblastoma, associated with histiocytosis-X and mosaicism of a deleted D-group chromosome (13q14 leads to q31). Clin Genet. 1982;22:37-9.

Oudesluijs GG, HUlzebos CV, Sikkema-Raddatz B, Van Essen AJ. Mosaic isodicentric chromosome 18q: sixth report and review. Genet Couns. 2006;17:395-400.

Õ[unap K](http://www.ncbi.nlm.nih.gov/pubmed/?term=OunapK%5BAuthor%5D&cauthor=true&cauthor_uid=15793836), [Ilus T](http://www.ncbi.nlm.nih.gov/pubmed/?term=IlusT%5BAuthor%5D&cauthor=true&cauthor_uid=15793836), [Bartsch O](http://www.ncbi.nlm.nih.gov/pubmed/?term=BartschO%5BAuthor%5D&cauthor=true&cauthor_uid=15793836). A girl with inverted triplication of chromosome 3q25.3 --> q29 and multiple congenital anomalies consistent with 3q duplication syndrome. [Am J Med Genet.](http://www.ncbi.nlm.nih.gov/pubmed/15793836) 2005;134 A:434-8.

Pabst B, Miller K, Bohnhorst B, Weidemann J, Schmidtke J, Arslan-Kirchner M. Mosaic interstitial duplication of the long arm of chromosome 20 associated with vertebral malformations as the only major phenotypic manifestation. Eur J Hum Genet. 2012;20 Suppl 1:105.

Pagon RA, Hall JG, Davenport SL, Aase J, Norwood TH, Hoehn HW. Abnormal skin fibroblast cytogenetics in four dysmorphic patients with normal lymphocyte chromosomes. Am J Hum Genet. 1979;31:54-61.

[Palka C](http://www.ncbi.nlm.nih.gov/pubmed/?term=PalkaC%5BAuthor%5D&cauthor=true&cauthor_uid=22144704), [Alfonsi M](http://www.ncbi.nlm.nih.gov/pubmed/?term=AlfonsiM%5BAuthor%5D&cauthor=true&cauthor_uid=22144704), [Mohn A](http://www.ncbi.nlm.nih.gov/pubmed/?term=MohnA%5BAuthor%5D&cauthor=true&cauthor_uid=22144704), [Cerbo R](http://www.ncbi.nlm.nih.gov/pubmed/?term=CerboR%5BAuthor%5D&cauthor=true&cauthor_uid=22144704), [Guanciali Franchi P](http://www.ncbi.nlm.nih.gov/pubmed/?term=GuancialiFranchiP%5BAuthor%5D&cauthor=true&cauthor_uid=22144704), [Fantasia D](http://www.ncbi.nlm.nih.gov/pubmed/?term=FantasiaD%5BAuthor%5D&cauthor=true&cauthor_uid=22144704), [Morizio E](http://www.ncbi.nlm.nih.gov/pubmed/?term=MorizioE%5BAuthor%5D&cauthor=true&cauthor_uid=22144704), [Stuppia L](http://www.ncbi.nlm.nih.gov/pubmed/?term=StuppiaL%5BAuthor%5D&cauthor=true&cauthor_uid=22144704), [Calabrese G](http://www.ncbi.nlm.nih.gov/pubmed/?term=CalabreseG%5BAuthor%5D&cauthor=true&cauthor_uid=22144704), [Zori R](http://www.ncbi.nlm.nih.gov/pubmed/?term=ZoriR%5BAuthor%5D&cauthor=true&cauthor_uid=22144704), [Chiarelli F](http://www.ncbi.nlm.nih.gov/pubmed/?term=ChiarelliF%5BAuthor%5D&cauthor=true&cauthor_uid=22144704), [Palka G](http://www.ncbi.nlm.nih.gov/pubmed/?term=PalkaG%5BAuthor%5D&cauthor=true&cauthor_uid=22144704). Mosaic 7q31 deletion involving FOXP2 gene associated with language impairment. [Pediatrics](http://www.ncbi.nlm.nih.gov/pubmed/22144704). 2012;129:e183-8.

Pallister PD, Patau K, Inhorn SL, Opitz JM. A woman with multiple congenital anomalies, mental retardation and mosaicism for an unusual translocation chromosome t(6;19). Clin Genet. 1974;5:188-95.

[Pangalos C](http://www.ncbi.nlm.nih.gov/pubmed/?term=PangalosC%5BAuthor%5D&cauthor=true&cauthor_uid=6609671), [Velissariou V](http://www.ncbi.nlm.nih.gov/pubmed/?term=VelissariouV%5BAuthor%5D&cauthor=true&cauthor_uid=6609671), [Ghica M](http://www.ncbi.nlm.nih.gov/pubmed/?term=GhicaM%5BAuthor%5D&cauthor=true&cauthor_uid=6609671), [Liacacos D](http://www.ncbi.nlm.nih.gov/pubmed/?term=LiacacosD%5BAuthor%5D&cauthor=true&cauthor_uid=6609671). Ring-14 and trisomy 14q in the same child. [Ann Genet](http://www.ncbi.nlm.nih.gov/pubmed/?term=pangalos+1984).  1984;27:38-40.

Parker CE, Alfi OS, Derencsenyi A, Mavalwala J, Donnell G. A child with a ring-4 chromosome (46,XX-46,XX,r 4). Am J Dis Child. 1974 ;128:371-4.

Paz-y-Mino C, Benitez J, Ayuso C, Sanchez-Cascos A. Ring chromosome 6: clinical and cytogenetic behaviour. Am J Med Genet. 1990 ;35:481-3.

Pellegrino JE, Schure RE, Kline R, Zackai EH, Spinner NB. Mosaic loss of 15q11q13 in a patient with hypomelanosis of Ito: is there a role for the P gene? Hum Genet. 1995;96:485-9.

Perfumo C, Cerruti Mainardi P, Cali A, Coucourde G, Zara F, Cavani S, Overhauser J, Bricarelli FD, Pierluigi M. The first three mosaic cri du chat syndrome patients with two rearranged cell lines. J Med Genet. 2000;37:967-72.

Petit P, Koulischer L. Report of a case of 46,XX/46,XX,17r mosaicism. Ann Genet. 1971;14:55-8.

Petty EM, Gibson LH, Breg WR, Burns JP, Yang-Feng TL. Mosaic dup (9p) diagnosed by fluorescence in situ hybridization (FISH). Am J Med Genet. 1993;45:770-3.

Pfeiffer RA, Dhadial R, Lenz W. 46,XX/46,XX,r(15) mosaicism: report of a case. J Med Genet. 1977;14:63-5.

Pfeiffer R, Lenard H. Ring chromosome 8(46,XY,8 r) in a boy with debility. Klin Pediatr. 1973;185:187-91.

Powell CM, Ellingham TJ, Rosenbaum KM, Stanley WS. Unbalanced 15;18 translocation in a Prader-Willi patient mosaic for a normal cell line. Am J Hum Genet. 1991;49 Suppl :261.

Powis Z, Kang S-H L, Cooper ML, Patel A, Peiffer DA, Hawkins A, Heidenreich R, Gunderson KL, Cheung SW, Erickson RP. Mosaic tetrasomy 12p with triplication of 12p detected by array-based comparative genomic hybridization of peripheral blood DNA. Am J Med Genet. 2007;143A:2910-5.

Putoux A, Labalme A, André J-M, Till M, Schluth-Bolard C, Berard J, Bertrand Y, Edery P, Putet G, Sanlaville D. Jacobsen and Beckwith-Wiedemann syndromes in a child with mosaicism for partial 11pter trisomy and partial 11qter monosomy. Am J Med Genet. 2013;161A:331-7.

Rahnama M, Hormozian F, Tootian S, Mortezapour F, et al. Mosaic Cri du Chat syndrome in a patient exhibiting two 5p cell lines. Chromosome Res. 2005;13 Suppl 1:76.

Rauch A, Trautmann U, Pfeiffer RA. Clinical and molecular cytogenetic observation in three cases of “trisomy 12p syndrome”. Am J Med Genet. 1996;63:243-9.

Rauen KA, Bitts SM, Li L, Golabi M, Cotter PD. Tandem duplication mosaicism: characterization of a mosaic dup(5q) and review. Clin Genet. 2001;60:366-70.

Receveur A, Bourel E, Andrieux J, Barathon M, Morin G, Amram F, Dery T, Jobic F, Jedraszak G, Mathieu-Dramard, Copin H, Demeer B. Mosaic ring chromosome 17: a new case. Eur J Hum Genet. 2014;22 Suppl 1:274.

Reddy KS, Larsen MB. A molecular, cytogenetic, and clinical evaluation of mosaic tandem duplication 17p and Charcot-Marie-Tooth type 1A neuropathy. J Med Genet. 1998;35:169-72.

Reddy KS, Logan JJ. Intrachromosomal triplications: molecular cytogenetic and clinical studies. [Clin Genet](http://www.ncbi.nlm.nih.gov/pubmed/11005147). 2000;58:134-41.

Reddy KS, Sulkova V. A tale of three mosaics. Medizinishe Genet. 1997;9 Suppl 2:300.

Reddy KS, Sulkova V, Young H, Blancato JK, Haddad BR. 1999. De novo mosaic add(3) characterized to be trisomy 14q31-qter using spectral karyotyping and subtelomeric probes. Am J Med Genet 84: 318-321.

Rethore MO, Noel B, Couturier J, Prieur M, Lafourcade J, Lejeune J. 1976. The r(22) syndrome. Apropos of 4 new cases. Ann Genet 19:111.

Rethore MO, Caille B, Huet de Barochez Y, de Blois MC, Ravel A, Lejeune J. 1984. Ring chromosome 14. II. A case report of r(14) mosaicism. The r(14) phenotype. Ann Genet 27:91-95.

Ribeiro MCM, Andrade JAD, Erwenne CL, Brunoni D. 1988. Bilateral retinoblastoma associated with 13q- mosaicism: possible manifestation of a germline mutation. Cancer Genet Cytogenet 32:169-175.

Riccardi VM, Labhard M, Marcus ES. Hemihypertrophy with contralateral 16q chromosomal deletion. Am J Hum Genet. 1977;29:91A.

Ritter CL, Steele MW, Wenger SL, Cohen B. Chromosome mosaicism in hypomelanosis of Ito. Am J Med Genet. 1990;35:14-7.

[Robberecht C](http://www.ncbi.nlm.nih.gov/pubmed/?term=Robberecht%20C%5BAuthor%5D&cauthor=true&cauthor_uid=22490612), [Voet T](http://www.ncbi.nlm.nih.gov/pubmed/?term=Voet%20T%5BAuthor%5D&cauthor=true&cauthor_uid=22490612), [Utine GE](http://www.ncbi.nlm.nih.gov/pubmed/?term=Utine%20GE%5BAuthor%5D&cauthor=true&cauthor_uid=22490612), [Schinzel A](http://www.ncbi.nlm.nih.gov/pubmed/?term=Schinzel%20A%5BAuthor%5D&cauthor=true&cauthor_uid=22490612), [de Leeuw N](http://www.ncbi.nlm.nih.gov/pubmed/?term=de%20Leeuw%20N%5BAuthor%5D&cauthor=true&cauthor_uid=22490612), [Fryns JP](http://www.ncbi.nlm.nih.gov/pubmed/?term=Fryns%20JP%5BAuthor%5D&cauthor=true&cauthor_uid=22490612), [Vermeesch J](http://www.ncbi.nlm.nih.gov/pubmed/?term=Vermeesch%20J%5BAuthor%5D&cauthor=true&cauthor_uid=22490612). Meiotic errors followed by two parallel postzygotic trisomy rescue events are a frequent cause of constitutional segmental mosaicism. [Mol Cytogenet](http://www.ncbi.nlm.nih.gov/pubmed/?term=mol+cytogenet+2012+5+19). 2012;5:19.

[Rocchi M](http://www.ncbi.nlm.nih.gov/pubmed/?term=Rocchi%20M%5BAuthor%5D&cauthor=true&cauthor_uid=6380829), [Cigui I](http://www.ncbi.nlm.nih.gov/pubmed/?term=Cigui%20I%5BAuthor%5D&cauthor=true&cauthor_uid=6380829), [Archidiacono N](http://www.ncbi.nlm.nih.gov/pubmed/?term=Archidiacono%20N%5BAuthor%5D&cauthor=true&cauthor_uid=6380829), [Pecile V](http://www.ncbi.nlm.nih.gov/pubmed/?term=Pecile%20V%5BAuthor%5D&cauthor=true&cauthor_uid=6380829), [Porcelli G](http://www.ncbi.nlm.nih.gov/pubmed/?term=Porcelli%20G%5BAuthor%5D&cauthor=true&cauthor_uid=6380829), [Filippi G](http://www.ncbi.nlm.nih.gov/pubmed/?term=Filippi%20G%5BAuthor%5D&cauthor=true&cauthor_uid=6380829). A young girl with ring(18) mosaicism: cytogenetic studies and PEPA mapping. Clin Genet. 1984;26:156-60.

Romano C, Ragusa RM, Scillato F, Greco D, Amato G, Barletta C. Phenotypic and phoniatric findings in mosaic cri du chat syndrome. Am J Med Genet. 1991;39:391-5.

[Rossi M](http://www.ncbi.nlm.nih.gov/pubmed/?term=Rossi%20M%5BAuthor%5D&cauthor=true&cauthor_uid=23165966), [Labalme A](http://www.ncbi.nlm.nih.gov/pubmed/?term=Labalme%20A%5BAuthor%5D&cauthor=true&cauthor_uid=23165966), [Cordier MP](http://www.ncbi.nlm.nih.gov/pubmed/?term=Cordier%20MP%5BAuthor%5D&cauthor=true&cauthor_uid=23165966), [Till M](http://www.ncbi.nlm.nih.gov/pubmed/?term=Till%20M%5BAuthor%5D&cauthor=true&cauthor_uid=23165966), [Blanchard G](http://www.ncbi.nlm.nih.gov/pubmed/?term=Blanchard%20G%5BAuthor%5D&cauthor=true&cauthor_uid=23165966), [Dubois R](http://www.ncbi.nlm.nih.gov/pubmed/?term=Dubois%20R%5BAuthor%5D&cauthor=true&cauthor_uid=23165966), [Guibaud L](http://www.ncbi.nlm.nih.gov/pubmed/?term=Guibaud%20L%5BAuthor%5D&cauthor=true&cauthor_uid=23165966), [Heissat S](http://www.ncbi.nlm.nih.gov/pubmed/?term=Heissat%20S%5BAuthor%5D&cauthor=true&cauthor_uid=23165966), [Javouhey E](http://www.ncbi.nlm.nih.gov/pubmed/?term=Javouhey%20E%5BAuthor%5D&cauthor=true&cauthor_uid=23165966), [Lachaux A](http://www.ncbi.nlm.nih.gov/pubmed/?term=Lachaux%20A%5BAuthor%5D&cauthor=true&cauthor_uid=23165966), [Mure PY](http://www.ncbi.nlm.nih.gov/pubmed/?term=Mure%20PY%5BAuthor%5D&cauthor=true&cauthor_uid=23165966), [Ville D](http://www.ncbi.nlm.nih.gov/pubmed/?term=Ville%20D%5BAuthor%5D&cauthor=true&cauthor_uid=23165966), [Edery P](http://www.ncbi.nlm.nih.gov/pubmed/?term=Edery%20P%5BAuthor%5D&cauthor=true&cauthor_uid=23165966), [Sanlaville D](http://www.ncbi.nlm.nih.gov/pubmed/?term=Sanlaville%20D%5BAuthor%5D&cauthor=true&cauthor_uid=23165966). Mosaic 18q21.2 deletions including the TCF4 gene: a clinical report. [Am J Med Genet.](http://www.ncbi.nlm.nih.gov/pubmed/?term=am+j+med+genet+2012+158a+12+3174)  2012;158A:3174-81.

Rouder JN, Speckman PL, Sun D, Morey RD, Iverson G. Bayesian t-tests for accepting and rejecting the null hypothesis. Psychonom Bull & Review. 2009;16:225‑37.

Rudnik-Schoneborn S, Schubert R, Majewski F, Haverkamp F, Schwanitz G. Partial trisomy 6p from a de novo translocation (6;18) with variable mosaicism in different tissues. Clin Genet. 1997;52:126-9.

Şamli H, Özgöz A, Mutlu Içduygu F, Hekimler K., İmirzalıoğlu N, Sıvacı Y. A case with mosaic ring chromosome 18. Eur J Hum Genet. 2009;17 Suppl 2:125.

Saura R, Longy M, Serville F, Chokairi O, Froute MF. Abnormal phenotype in a child with a "balanced" translocation 8/12 in mosaic state. Am J Med Genet. 1987 ;28:1021-3.

Sauter S, von Beust G, Burfeind P, Weise A, Starke H, Liehr T, Zoll B. Autistic disorder and chromosomal mosaicism 46,XY[123]/46,XY,del(20)(pter -> p12.2)[10]. Am J Med Genet. 2003;120A:533-6.

Say B, Carpenter NJ. Report of a case resembling the Proteus syndrome with a chromosome abnormality. Am J Med Genet. 1988;31:987-9.

[Schluth C](http://www.ncbi.nlm.nih.gov/pubmed/?term=SchluthC%5BAuthor%5D&cauthor=true&cauthor_uid=15940678), [Mattei MG](http://www.ncbi.nlm.nih.gov/pubmed/?term=MatteiMG%5BAuthor%5D&cauthor=true&cauthor_uid=15940678), [Mignon-Ravix C](http://www.ncbi.nlm.nih.gov/pubmed/?term=Mignon-RavixC%5BAuthor%5D&cauthor=true&cauthor_uid=15940678), [Salman S](http://www.ncbi.nlm.nih.gov/pubmed/?term=SalmanS%5BAuthor%5D&cauthor=true&cauthor_uid=15940678), [Alembik Y](http://www.ncbi.nlm.nih.gov/pubmed/?term=AlembikY%5BAuthor%5D&cauthor=true&cauthor_uid=15940678), [Willig J](http://www.ncbi.nlm.nih.gov/pubmed/?term=WilligJ%5BAuthor%5D&cauthor=true&cauthor_uid=15940678), [Ginglinger E](http://www.ncbi.nlm.nih.gov/pubmed/?term=GinglingerE%5BAuthor%5D&cauthor=true&cauthor_uid=15940678), [Jeandidier E](http://www.ncbi.nlm.nih.gov/pubmed/?term=JeandidierE%5BAuthor%5D&cauthor=true&cauthor_uid=15940678). Intrachromosomal triplication for the distal part of chromosome 15q. [Am J Med Genet.](http://www.ncbi.nlm.nih.gov/pubmed/15940678)  2005;136A:179-84.

Schmidt H, Uhrig S, Lederer G, Murken J, Schuffenhauer S. Mosaicism for a dup(12)(q22q13) in a patient with hypomelanosis of Ito and asymmetry. J Med Genet. 2000;37:804-6.

[Schwartz S](http://www.ncbi.nlm.nih.gov/pubmed/?term=SchwartzS%5BAuthor%5D&cauthor=true&cauthor_uid=1778004), [Harris M](http://www.ncbi.nlm.nih.gov/pubmed/?term=HarrisM%5BAuthor%5D&cauthor=true&cauthor_uid=1778004), [Ehrenpreis R](http://www.ncbi.nlm.nih.gov/pubmed/?term=EhrenpreisR%5BAuthor%5D&cauthor=true&cauthor_uid=1778004), [Zaslav A](http://www.ncbi.nlm.nih.gov/pubmed/?term=ZaslavA%5BAuthor%5D&cauthor=true&cauthor_uid=1778004), [Raffel LJ](http://www.ncbi.nlm.nih.gov/pubmed/?term=RaffelLJ%5BAuthor%5D&cauthor=true&cauthor_uid=1778004), [Schwartz MF](http://www.ncbi.nlm.nih.gov/pubmed/?term=SchwartzMF%5BAuthor%5D&cauthor=true&cauthor_uid=1778004), [Lieber E](http://www.ncbi.nlm.nih.gov/pubmed/?term=LieberE%5BAuthor%5D&cauthor=true&cauthor_uid=1778004), [Cohen MM](http://www.ncbi.nlm.nih.gov/pubmed/?term=CohenMM%5BAuthor%5D&cauthor=true&cauthor_uid=1778004). De novo 13q partial duplication identified by cytogenetic, biochemical and molecular approaches. [Clin Genet](http://www.ncbi.nlm.nih.gov/pubmed/1778004). 1991;40:417-22.

Serotkin A, Stamberg J, Waber L. Duplication 17q mosaicism: an infant with features of Ellis-van Creveld syndrome. J Med Genet. 1988;25:258-60.

Shaham M, Beachman S, Vogel J, McGivans M, Carnahan A, Chaban PA, Searle BM. Mosaicism for balanced and unbalanced structural chromosome rearrangements. Am J Hum Genet. 1992;51Suppl:A296.

Shanske AL, Patel A, Saukam S, Levy B, Lüdecke HJ. Clinical and molecular characterization of a patient with Langer-Giedion syndrome and mosaic del(8)(q22.3q24.13). Am J Med Genet. 2008;146A:3211-6.

Shapira SK, Kochanek S, Shaffer LG. A rare mosaic interstitial deletion of 7q, 46,XX/46,XX,del(7)(q22.1q31.33). Am J Hum Genet. 1994;55 Suppl 3:A118.

Smith A, den Dulk G, Viersbach R, Michas J. Ring chromosome 15 and 15qs+ mosaic: clinical and cytogenetic behaviour spanning 29 years. Am J Med Genet. 1991;40:460-3.

Sotnikova EN, Zaletayev DV. 1991. A case of mosaic deletion of chromosome 13 in a patient with rethinoblastoma. In: Kuleshov NP, Lourie IW, editors. Current problems in clinical cytogenetics, Moskow: 1991. p. 64.

[Soysal Y](http://www.ncbi.nlm.nih.gov/pubmed/?term=Soysal%20Y%5BAuthor%5D&cauthor=true&cauthor_uid=19921639), [Balci S](http://www.ncbi.nlm.nih.gov/pubmed/?term=Balci%20S%5BAuthor%5D&cauthor=true&cauthor_uid=19921639), [Hekimler K](http://www.ncbi.nlm.nih.gov/pubmed/?term=Hekimler%20K%5BAuthor%5D&cauthor=true&cauthor_uid=19921639), [Liehr T](http://www.ncbi.nlm.nih.gov/pubmed/?term=Liehr%20T%5BAuthor%5D&cauthor=true&cauthor_uid=19921639), [Ewers E](http://www.ncbi.nlm.nih.gov/pubmed/?term=Ewers%20E%5BAuthor%5D&cauthor=true&cauthor_uid=19921639), [Schoumans J](http://www.ncbi.nlm.nih.gov/pubmed/?term=Schoumans%20J%5BAuthor%5D&cauthor=true&cauthor_uid=19921639), [Bui TH](http://www.ncbi.nlm.nih.gov/pubmed/?term=Bui%20TH%5BAuthor%5D&cauthor=true&cauthor_uid=19921639), [Içduygu FM](http://www.ncbi.nlm.nih.gov/pubmed/?term=Içduygu%20FM%5BAuthor%5D&cauthor=true&cauthor_uid=19921639), [Kosyakova N](http://www.ncbi.nlm.nih.gov/pubmed/?term=Kosyakova%20N%5BAuthor%5D&cauthor=true&cauthor_uid=19921639), [Imirzalioğlu N](http://www.ncbi.nlm.nih.gov/pubmed/?term=Imirzalioğlu%20N%5BAuthor%5D&cauthor=true&cauthor_uid=19921639). Characterization of double ring chromosome 4 mosaicism associated with bilateral hip dislocation, cortical dysgenesis, and epilepsy. [Am J Med Genet.](http://www.ncbi.nlm.nih.gov/pubmed/?term=am+j+med+genet+2009+149a+2782)  2009;149A:2782-7.

Srcen S, Volna J, Miklerova M. A mosaic of ring chromosome 18. Cesk Pediatr. 1980;35:419-22.

Streubel B, Latta E, Kehrer-Sawatzki H, Hoffman GF, Fonatsch C, Rehder H. Somatic mosaicism of a greater than 1.7-Mb deletion of genomic DNA involving the entire NF1 gene as verified by FISH: Further evidence for a contriguous gene syndrome in 17q11.2. Am J Med Genet. 1999;87:12-6.

Sukarova-Angelovska E, Kocova M, Angelkova N, Ilieva G. Mild phenotype in a child with low rate mosaic 11q deletion. Eur J Hum Genet. 2007;15 Suppl 1:68.

Summitt RL, Tharapel AT, Wilroy RS. Tissue limited mosaicism for unbalanced autosomal translocation in a child with congenital anomalies and mental retardation. Eur J Pediatr. 1977;125:169-74.

Suskov II, Rubtsov GA. A case of mosaicism 46,XY/46,XY,3q+. The Register of chromosomal anomalies, Institute of Medical Genetics, Moskow: 1984. p. 73.

Sutherland GR, Carter RF. 46,XX/46,XX,r (2)(p25q37) mosaicism: clinical and cytogenetic studies. Ann Genet. 1978;21:164-7.

Sybert VP, Bradley CM, Salk D. Mosaicism for ring 19: a case report. Clin Genet. 1988;34:382-5.

[Takenouchi T](http://www.ncbi.nlm.nih.gov/pubmed/?term=TakenouchiT%5BAuthor%5D&cauthor=true&cauthor_uid=22887345), [Yagihashi T](http://www.ncbi.nlm.nih.gov/pubmed/?term=YagihashiT%5BAuthor%5D&cauthor=true&cauthor_uid=22887345), [Tsuchiya H](http://www.ncbi.nlm.nih.gov/pubmed/?term=TsuchiyaH%5BAuthor%5D&cauthor=true&cauthor_uid=22887345), [Torii C](http://www.ncbi.nlm.nih.gov/pubmed/?term=ToriiC%5BAuthor%5D&cauthor=true&cauthor_uid=22887345), [Hayashi K](http://www.ncbi.nlm.nih.gov/pubmed/?term=HayashiK%5BAuthor%5D&cauthor=true&cauthor_uid=22887345), [Kosaki R](http://www.ncbi.nlm.nih.gov/pubmed/?term=KosakiR%5BAuthor%5D&cauthor=true&cauthor_uid=22887345), [Saitoh S](http://www.ncbi.nlm.nih.gov/pubmed/?term=SaitohS%5BAuthor%5D&cauthor=true&cauthor_uid=22887345), [Takahashi T](http://www.ncbi.nlm.nih.gov/pubmed/?term=TakahashiT%5BAuthor%5D&cauthor=true&cauthor_uid=22887345), [Kosaki K](http://www.ncbi.nlm.nih.gov/pubmed/?term=KosakiK%5BAuthor%5D&cauthor=true&cauthor_uid=22887345). Tissue-limited ring chromosome 18 mosaicism as a cause of Pitt-Hopkins syndrome. [Am J Med Genet.](http://www.ncbi.nlm.nih.gov/pubmed/22887345)  2012;158A:2621-3.

[Tekin M](http://www.ncbi.nlm.nih.gov/pubmed/?term=TekinM%5BAuthor%5D&cauthor=true&cauthor_uid=11078565), [Jackson-Cook C](http://www.ncbi.nlm.nih.gov/pubmed/?term=Jackson-CookC%5BAuthor%5D&cauthor=true&cauthor_uid=11078565), [Buller A](http://www.ncbi.nlm.nih.gov/pubmed/?term=BullerA%5BAuthor%5D&cauthor=true&cauthor_uid=11078565), [Ferreira-Gonzalez A](http://www.ncbi.nlm.nih.gov/pubmed/?term=Ferreira-GonzalezA%5BAuthor%5D&cauthor=true&cauthor_uid=11078565), [Pandya A](http://www.ncbi.nlm.nih.gov/pubmed/?term=PandyaA%5BAuthor%5D&cauthor=true&cauthor_uid=11078565), [Garrett CT](http://www.ncbi.nlm.nih.gov/pubmed/?term=GarrettCT%5BAuthor%5D&cauthor=true&cauthor_uid=11078565), [Bodurtha J](http://www.ncbi.nlm.nih.gov/pubmed/?term=BodurthaJ%5BAuthor%5D&cauthor=true&cauthor_uid=11078565). Fluorescence in situ hybridization detectable mosaicism for Angelman syndrome with biparental methylation. [Am J Med Genet](http://www.ncbi.nlm.nih.gov/pubmed/11078565). 2000;95:145-9.

Tengstrom C, Autio S. Chromosomal aberrations in 85 mentally retarded patients examined by high resolution banding. Clin Genet. 1987;31:53-60.

Théophile D, de Blois MC, Barth D, Gilbert B, Picq M, Delabar J, Prieur M, Vekemans M. Partial correction of monosomy 21 by a duplication of chromosome 21. Am J Hum Genet. 1993 ;53 Suppl 3:610.

Thapa R, Bhattacharya S, Ghosh A. Mental retardation and developmental delay due to mosaic chromosome 14q duplication. J Dev Behav Pediatr. 2008;29:541-2.

Thomas IT, Frias J, Cantu ES, Lafer CZ, Flannery DB, Graham JG. Association of pigmentary anomalies with chromosomal and genetic mosaicism and chimerism. Am J Hum Genet. 1989;45:193-205.

Tsukamoto H, Inui K, Taniike M, Kamiyama K, Hori M, Sumi K, Okada S. Different clinical features in monozygotic twins: a case of 7q--syndrome. Clin Genet. 1993;43:139-42.

Tunca Y, Wilroya RS, Kadandaleb JS, Martensb PR, Guntherc WM, Tharapel AT. Hypomelanosis of Ito and a ‘mirror image’ whole chromosome duplication resulting in trisomy 14 mosaicism. Ann Genet. 2000;43:39-43.

Turleau C, Rethore MO, Junien C, Lejeune J, de Grouchy J. 46,XX/46,XX,del (10) (p13)/47,XX,+r/47,XX,del (10) (p13), + r mosaicism and partial trisomy 10p phenotype. Ann Genet. 1979 ;22:178-81.

Turleau C, Taillard F, Doussau de Bazignan M, Delepine N, Desbois JC, de Grouchy J. Hypomelanosis of Ito (incontinentia pigmenti achromians) and mosaicism for a microdeletion of 15q. Hum Genet. 1986 ;74:185-7.

Tuzun C, Ocal B, Karamollaoglo K, Altintas K, Ensari C, Demireller T, Azkin H. A case with 46,XX/46,XX,del(11)(q24). Medizinishe Genet. 1997;9 Suppl 2:302.

Uliana V, Biancheri R, Doria Lambda L, Rossi A, Severino M, Malacarne M, Marciano C, Mandrile G, Forzano F, Di Maria E, Faravelli F. Diagnostic pitfalls and mosaic unbalanced translocations: a case of 18q- deletion syndrome Eur J Hum Genet. 2013;21 Suppl 2:480-1.

Utine GE, Aktas D. 2006. Mosaicism for terminal deletion of 4q. Genet Couns 17:205-209.

van Haelst M, Bhat M, Bint S. Mosaic de novo interstitial duplication of chromosome 17q. J Med Genet. 2005;42 Suppl 1:S53.

Vargas MT, Fernandez-Novoa MC. Balanced reciprocal translocation mosaicism: clinical implications. Two new cases. Genet Couns. 2001;12:269-71.

[Vecchi M](http://www.ncbi.nlm.nih.gov/pubmed/?term=VecchiM%5BAuthor%5D&cauthor=true&cauthor_uid=21893419), [Cassina M](http://www.ncbi.nlm.nih.gov/pubmed/?term=CassinaM%5BAuthor%5D&cauthor=true&cauthor_uid=21893419), [Casarin A](http://www.ncbi.nlm.nih.gov/pubmed/?term=CasarinA%5BAuthor%5D&cauthor=true&cauthor_uid=21893419), [Rigon C](http://www.ncbi.nlm.nih.gov/pubmed/?term=RigonC%5BAuthor%5D&cauthor=true&cauthor_uid=21893419), [Drigo P](http://www.ncbi.nlm.nih.gov/pubmed/?term=DrigoP%5BAuthor%5D&cauthor=true&cauthor_uid=21893419), [De Palma L](http://www.ncbi.nlm.nih.gov/pubmed/?term=DePalmaL%5BAuthor%5D&cauthor=true&cauthor_uid=21893419), [Clementi M](http://www.ncbi.nlm.nih.gov/pubmed/?term=ClementiM%5BAuthor%5D&cauthor=true&cauthor_uid=21893419). Infantile epilepsy associated with mosaic 2q24 duplication including SCN2A and SCN3A. [Seizure](http://www.ncbi.nlm.nih.gov/pubmed/21893419). 2011;20:813-6.

Vermeesch JR, Syrrou M, Salden I, Dhondt F, Matthijs G, Fryns JP. Mosaicism for duplication 12q (12q13-->12q21.2) accompanied by a pericentric inversion in a dysmorphic female infant. J Med Genet. 2002;39:e72.

Vigfusson NV, Kapstafer KJ, Lloyd MA. Ring chromosome 2 in a child with growth failure and few congenital abnormalities. Am J Med Genet. 1980;7:383-9.

Vockley J, Inserra J, Berg RW, Yang-Feng TL. Pseudomosaicism for 4p- in amniotic fluid cell culture proven to be true mosaicism after birth. Am J Med Genet. 1991;39:81-3.

Wilmot PL, Shapiro LR, Brenholz P, Leff A, Neier S, Martino M. Structural chromosome mosaicism: incidence among 3500 cytogenetic analyses. Am J Hum Genet. 1982;34 Suppl 6:150A.

Woods CG, Bankier A, Curry J, Sheffield LJ, Slaney SF, Smith K, Voullaire L, Wellesley D. Asymmetry and skin pigmentary anomalies in chromosome mosaicism. J Med Genet. 1994;31:694-701.

Wyandt HE, Huang XL, Milunsky JM. Partial trisomy 16q in a full term infant, mosaic for an unbalanced translocation, der(19)t(16;19)(q13;q13). Am J Hum Genet. 1999;65 Suppl 4:2052.

Yip MY, Parsons A, Hulten M. A de novo tandem duplication 15(q21-qter) mosaic. Clin Genet. 1982;22:1-6.

Yong YP, Knight LA, Yong MH, Lam S, Ho LY. Partial monosomy for chromosome 22 in a girl with mental retardation. Singapore Med J. 1997;38:85-6.

Yung JF, Sobel DB, Hoo JJ. Origin of 46,XY/46,XY,r(19) mosaicism. Am J Med Genet. 1990;36:391-3.

Zaletayev DV, Kuleshov NP, Lurie IV, Fedotov VP. 1984. A case of Langer-Gideon as a result of interstitial deletion del(8)(q2411q24.13). In: Kuleshov NP and Lurie IW (eds.), The Register of chromosomal anomalies. Institute of Medical Genetics, Moskow, p.115-116.

Zaslav AL, Fallet S, Blumenthal D, Jacob J, Fox J. 1999. Mosaicism with a normal cell line and an unbalanced structural rearrangement. Am J Med Genet 82:15-19.
